# Immunoproteasome Inhibition Impairs T and B Cell Activation by Restraining ERK Signaling and Proteostasis

**DOI:** 10.3389/fimmu.2018.02386

**Published:** 2018-10-26

**Authors:** Christian Schmidt, Thilo Berger, Marcus Groettrup, Michael Basler

**Affiliations:** ^1^Chair of Immunology, Department of Biology, University of Konstanz, Konstanz, Germany; ^2^Konstanz Research School Chemical Biology, University of Konstanz, Konstanz, Germany; ^3^Biotechnology Institute Thurgau at the University of Konstanz, Kreuzlingen, Switzerland

**Keywords:** immunoproteasome, ONX 0914, ERK, proteostasis, T cell activation, B cell activation, Nrf1, DUSP6

## Abstract

Immunoproteasome (IP) inhibition holds potential as a novel treatment option for various immune-mediated pathologies. The IP inhibitor ONX 0914 reduced T cell cytokine secretion and Th17 polarization and showed pre-clinical efficacy in a range of autoimmune disorders, transplant-allograft rejection, virus-mediated tissue damage, and colon cancer progression. However, the molecular basis of these effects has remained largely elusive. Here, we have analyzed the effects of ONX 0914 in primary human and mouse lymphocytes. ONX 0914-treatment impaired primary T cell activation *in vitro* and *in vivo*. IP inhibition reduced ERK-phosphorylation sustainment, while leaving NF-κB and other signaling pathways unaffected. Naïve T and B cells expressed nearly exclusively immuno- or mixed proteasomes but no standard proteasomes and IP inhibition but not IP-deficiency induced mild proteostasis stress, reduced DUSP5 expression and enhanced DUSP6 protein levels due to impaired degradation. However, accumulation of DUSP6 did not cause the reduced ERK-phosphorylation in a non-redundant manner. We show that broad-spectrum proteasome inhibition and immunoproteasome inhibition have distinct effects on T cell activation at the molecular level. Notably, ONX 0914-treated T cells recovered from proteostasis stress without apoptosis induction, apparently via Nrf1-mediated up-regulation of standard proteasomes. In contrast, B cells were more susceptible to apoptosis after ONX 0914-treatment. Our data thus provide mechanistic insights how IP inhibition functionally impedes T and B cells likely accounting for its therapeutic benefits.

## Introduction

Immunoproteasomes (IP) constitute a specialized form of proteasome expressed in leukocytes and in inflamed tissue (1–4). Upon stimulation of cells with interferon (IFN)-γ, three inducible β-subunits substitute the subunits β1c, β2c, and β5c of the standard proteasome during *de novo* assembly (5–8). Low molecular mass polypeptide (LMP)2 incorporates for β1c, multicatalytic endopeptidase complex-like (MECL)-1 substitutes for β2c, and LMP7 incorporates at the β5c position, leading to well characterized changes in peptidolytic cleavage priorities (9). IPs are well characterized for their involvement in MHC-I antigen processing (9–11). Antigen processing independent functions have recently been found in studies using immunoproteasome-subunit-deficient mice or IP inhibitors (12–15). However, to which extent and by which molecular mechanism IPs play such a role for immune and non-immune cells at steady state or during inflammation has remained controversial (16–18).

Several pre-clinical studies showed beneficial effects of IP inhibition in both primarily T cell-mediated auto-immune disease models like experimental autoimmune encephalomyelitis, rheumatoid arthritis, inflammatory bowel disease as well as antibody-linked disorders like systemic lupus erythematosus and experimental myasthenia gravis (19–25). Recently, IP inhibition also showed efficacy in preventing allograft rejection after kidney transplantation (26), reduced inflammation after cardiac allograft transplantation (27), attenuated colon cancer progression (28, 29), and protected from virus-mediated severe myocarditis (30). Furthermore, proteasome inhibitors are clinically used for the treatment of multiple myeloma, but side effects limit their broader applicability (31).

Since its original description as an LMP7-selective inhibitor, the molecular mechanism by which ONX 0914 affects the progression of auto-immune pathologies has remained elusive. Here, we characterized the effect of ONX 0914-treatment on activation of primary human and murine T and B cells which to our surprise almost exclusively expressed immunoproteasomes and barely any standard proteasome. IP inhibition but not genetic ablation of LMP7 blunted ERK-signaling sustainment and induced mild proteostasis stress, thereby differentially affecting T and B lymphocyte function and survival.

## Materials and methods

Additional information on method details and key resources are provided in the Supplementary Material.

### Animals

C57BL/6J (H-2^b^) mice were originally purchased from Charles River. LMP7^−/−^ (10), and LMP2^−/−^ (32) mice were kindly provided by John J. Monaco (Cincinnati Medical Center, Cincinnati, USA). SMARTA mice (33) (SM1-Ly5.1) were provided by the Swiss Immunological Mutant Mouse Repository. DUSP6^−/−^ mice (34) were purchased from Charles River. LCMV-infection was performed as described previously (1). Animals were kept in an SPF environment in the Animal Facility at the University of Konstanz. Animal experiments were approved by the review board of Regierungspräsidium Freiburg (G-16/154, T-16/15TFA, and T-18/03TFA).

### Human voluntary donors

Peripheral blood was obtained from healthy voluntary human donors. Age and sex were unknown to the experimental investigator. Blood donations were provided in cooperation with Biotechnology Institute Thurgau (BITg), Kreuzlingen, Switzerland. The ethical committee of Kanton Thurgau, Switzerland, approved the blood donations and volunteers gave their informed consent.

### Cell isolation, culture, and activation

Splenic murine lymphocytes were isolated with CD19 beads, CD4+ T cell isolation kit or CD4 beads (Miltenyi) according to the manufacturer's protocol and cultured in RPMI 1640 +supplements. T cells were activated with plate-bound anti-CD3/anti-CD28 (Biolegend). Mouse IL-2 ELISA Ready-Set Go! (ebioscience) was used according to the manufacturer's protocol. For *ex vivo* expansion T cells were activated with PMA/ionomycin overnight, followed by cultivation in IL-2-containing medium for 6 days. B cells were activated with PMA/ionomycin or anti-CD40 (Biolegend) and F(ab')2 anti-mouse IgG (eBioscience). B cells were activated with 50 ng/ml PMA and 500 ng/ml ionomycin or 5 μg/ml anti-CD40 (Biolegend) and 10 μg/ml F(ab')2 anti-mouse IgG (eBioscience). T1 cells (35) were kindly provided by Wolgang Schamel, University of Freiburg, Germany, and cultured in RPMI 1640 +supplements. Human T cells were isolated from PBMCs of healthy volunteers according to the Miltenyi human CD4+ T cell isolation protocol and cultured in AIM-V medium +supplements. Cells were activated with the Human T cell activation and expansion kit (Miltenyi) according to the manufacturer's protocol.

### Immunoblotting

Lysates were generated with whole cell lysis buffer on ice. Insoluble debris was pelleted and discarded. Lysates were boiled in SDS-sample-buffer and stored at −20°C. Equal volumes were separated by SDS-PAGE (8–15%) and blotted onto nitrocellulose membranes (GE Healthcare). For ECL-based detection, membranes were blocked with 3% BSA in TBS-T and antibodies were diluted in 3% BSA in TBS-T (primary Ab overnight, 4°C, secondary for 1–3 h, RT). HRP-coupled anti-mouse/anti-rabbit secondary antibodies were purchased from Dako. Near-infrared detection was performed according to the LI-COR protocol. Secondary antibodies: IRDye800CW goat anti-rabbit or anti-mouse and IRDye680RD goat anti-mouse or anti-rabbit (1:15,000). Signals were quantified with the LI-COR Odyssey Imager and Image Studio Lite Vers.5.2.

### Radioactive labeling and immunoprecipitation

IFN-γ-stimulated T1 cells were pre-treated with inhibitors or DMSO, activated with plate-bound antibodies for 2–3 h, starved in cys/met-free medium for 1 h followed by 15 min radioactive labeling with ^35^S-cys/met. After washing lysates were used for immunoprecipitation against DUSP6 after 0, 20, and 40 min. Further details are outlined in the Supplementary Material.

### Confocal microscopy

Expanded CD4+ T cells were activated on poly-L-lysine and anti-CD3/-CD28-antibody coated glass coverslips, fixed with 4% PFA and stained against p-ERK1/2 or total ERK1/2 and DAPI. Images were obtained with a Zeiss LSM 880 and digitally analyzed using Fiji with macros as outlined in the Supplementary Material details.

### Flow cytometry

Surface staining was performed with antibodies diluted in FACS-buffer (20 min, 4°C). Intracellular staining was performed after 4% PFA fixation (5 min, 37°C), followed by surface staining, fixation/permeabilization in 90% 4°C-cold methanol (45 min), primary antibody staining at 4°C overnight in PERM-buffer and secondary Ab staining in PERM-buffer (2 h, 4°C). Analysis was performed with FACSFortessa, FACSCalibur, or C6Accuri instruments (BD) and FlowJo V10 software.

### Quantitative real-time PCR

RNA was isolated from −80°C frozen cell pellets (QIAGEN RNeasy Mini kit). After purity assessment with a NanoVue instrument (GE Healthcare) cDNA was synthesized using Oligo-dT-primers (Promega). q-RT-PCR (Roche FastStart DNA SYBR green-I kit) was performed in a Biometra TProfessional Thermocycler (Analytik Jena). Primers were designed with intron-exon-overlap and amplicons tested via melting curve and/or agarose gels. *Rpl13a* and *Ipo8* served as housekeeping genes. Data was analyzed with the 2^−ΔΔ*Ct*^ method (36).

### CFSE-proliferation assay

Up to 1 × 10^7^ cells were stained with 1 μM CFSE in 1 ml PBS for 10 min, washed twice with PBS and twice with medium. Cells were activated with plate-bound antibodies in the presence of ONX 0914 or DMSO. CFSE dilution was measured by flow cytometry after 72 h.

### Proteasome inhibitors

ONX 0914 and PR-825 were kindly provided by Christopher J. Kirk (Kezar Life Sciences, South San Francisco, USA) and stored at −80°C in DMSO. MG-132 (Sigma-Aldrich) was stored at −20°C in DMSO. For *in vivo* application, ONX 0914 was dissolved in Captisol (Ligand) as previously described (20).

### Quantification and statistical analysis

One-Sample *t*-test was performed for ratio values for compound-treated samples/DMSO-control-treated samples, null-hypothesis μ_0_ = 1, plotted as mean with 95%CI. Means from repeated experiments with more than two groups were subjected to repeated-measures ANOVA, Sidak's multiple comparison. Regular ANOVA was performed for analysis of values from mice in one experiment. Paired *t*-tests were performed for two-group comparisons from repeated experiments. Tests and graphical representation are outlined in figure legends. All statistical evaluation was computed with GraphPad Prism6 and significance levels are indicated in figures. Asterisks mark ^***^p < 0.001, ^**^p < 0.01, ^*^p < 0.05. A detailed description of statistical test choice and sample size is attached in the Supplementary Material.

## Results

### IP inhibition, but not deficiency, impaired early T cell activation

**Figure 1:**
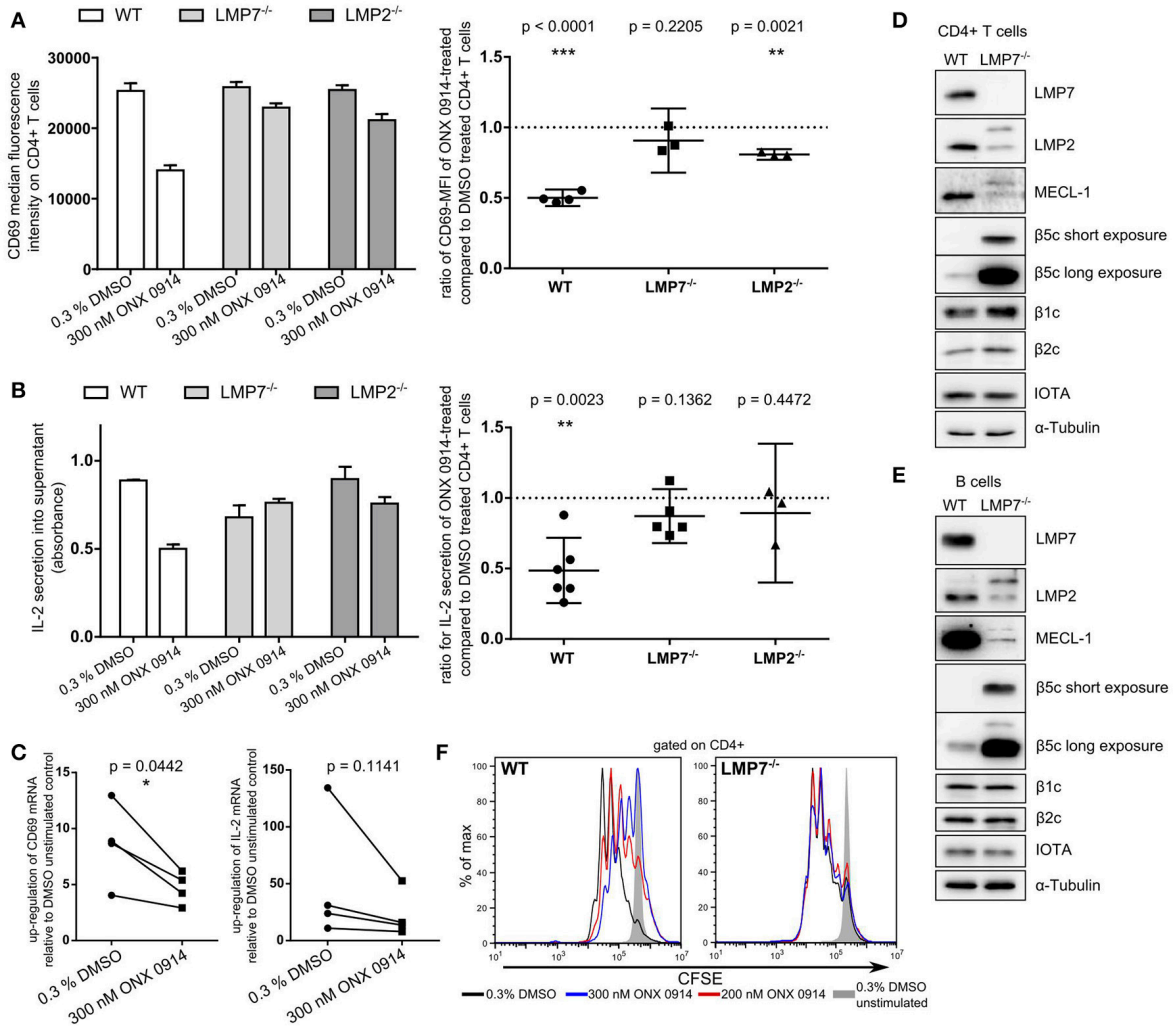
Immunoproteasome inhibition impairs T cell activation in an LMP7-/LMP2-co-dependent manner. **(A)** Purified naive T cells from WT, LMP7^−/−^, or LMP2^−/−^ mice were pulse-treated with DMSO or ONX 0914 for 2 h prior to activation with plate-bound anti-CD3/anti-CD28 antibodies for 5 h. CD69 MFI was measured on CD4+ cells by flow cytometry. Bar graph shows one representative example. Right hand graphs show ratios of ONX 0914-treated/DMSO-treated means from independent reproduction as mean + 95% CI for each genotype, *p*-values of one-sample *t*-tests. Horizontal dashed line indicates μ_0_. **(B)** Quantification of IL-2 secretion by ELISA from supernatants of cells purified and treated as in A. One representative example (bar graph) and ratios of ONX 0914/DMSO treated of three or more independent experiments represented as in **A**. **(C)** Purified WT CD4+ T cells were pulse-treated as in A and activated for 4 h. Total RNA was extracted and retro-transcribed cDNA was subjected to q-RT PCR for *Cd69* and *Il-2* transcripts. Shown is the fold up-regulation over unstimulated controls normalized to *Rpl13a*. *P*-values of paired *t*-tests as indicated. **(D**,**E)** Purified naive CD4+ T cells **(D)** or CD19+ B cells **(E)** were subjected to analysis of (immuno-) proteasome subunit protein expression by immunoblotting. One example each of three independent experiments are shown, α-tubulin and proteasome subunit α1 (IOTA) were used as loading controls. **(F)** CFSE-dilution profile of WT or LMP7^−/−^ derived CD4+ T cells after 72 h of activation with plate-bound antibodies in presence or absence of indicated ONX 0914 concentrations.

IP inhibition by ONX 0914 reduces cytokine secretion and impairs T cell function (22). To understand these effects at the molecular level, we investigated IP inhibition in T cell activation within the first hours. We purified CD4+ T cells from WT, LMP7-deficient or LMP2-deficient mice and pulse-treated them for 2 h with ONX 0914 or DMSO before activation. Within 5 h of activation, ONX 0914-treated cells showed ~50% reduced CD69 up-regulation and IL-2 secretion in WT, but not in LMP7-deficient cells and only to a small extent in LMP2-deficient cells (Figures 1A,B). CD69 and IL-2 expression also showed a trend toward reduction at the mRNA level in ONX 0914-treated WT cells (Figure 1C). Immunoblot analysis of all catalytic immuno- and standard proteasome subunits revealed that both T and B cells from WT mice expressed almost exclusively LMP7 while β5c was hardly detectable (Figures 1D,E). The subunits β1c and β2c were only slightly reduced in WT compared to LMP7-deficient cells indicating that WT lymphocytes express immuno- and mixed proteasomes, but no standard proteasomes at the naïve state. In contrast, LMP7-deficient cells had high β5c levels and low incorporation of matured LMP2 and MECL-1, while their precursors accumulated showing the need for LMP7 for their incorporation into the IP (Figures 1D,E). Hence, LMP7-deficient CD4+ cells contained mainly standard proteasomes. Furthermore, TCR-activation-induced proliferation over 3 days in the presence of ONX 0914 was impaired in WT, but not in LMP7-deficient cells (Figure 1F), substantiating that ONX 0914 acted immuno-subunit-selectively. As all observed effects were absent in cells lacking immunoproteasomes, these effects were not caused by an impact of ONX 0914 on unknown off-targets. Notably, recombinant m-IL-2-supplementation was insufficient to rescue proliferation in the presence of ONX 0914 (Figure S1A in Supplementary Material). Irrespective of ONX 0914-treatment, LMP7-deficient cells behaved like WT cells in all experiments, showing that IPs were dispensable and that standard proteasomes could compensate for loss of IPs in early T cell activation. These results suggested that IP inhibition, but not deficiency, impaired activation via an early cell-intrinsic mechanism, possibly involving impaired TCR-induced signaling.

### Ameliorated T cell activation *in vivo*

**Figure 2:**
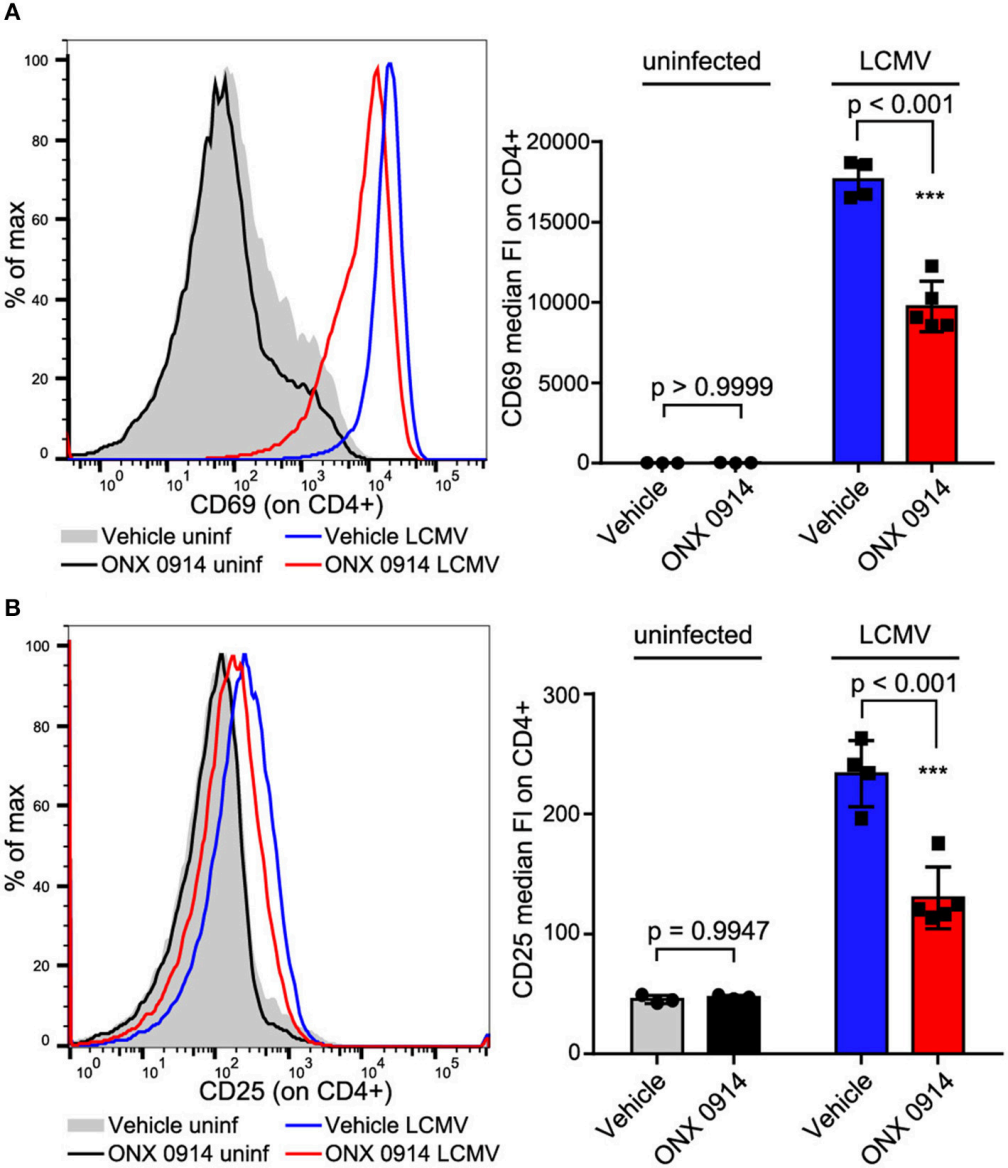
*In vivo* activated CD4+ T cells are impaired by ONX 0914. **(A,B)** SMARTA mice were treated with 10 mg/kg ONX 0914 or vehicle s.c. 2 h before i.v. infection with 4 × 10^5^ pfu LCMV or left uninfected. 18 h after infection, splenocytes were subjected to flow cytometry to assess CD69 and CD25 up-regulation on CD4+ T cells. Histograms show one representative profile of a mouse from each group. Bar graphs show pooled data from MFI quantifications (mean ± SD). Two-way ANOVA, Sidak's post-test, *p*-values as indicated in the figure; *n* = 3 for uninfected controls, *n* = 4 or 5 mice for infected group.

We aimed to test whether the impaired activation was similar for *in vivo* activated cells at efficacious doses applied in pre-clinical models. Therefore, we treated SMARTA mice (TCR-transgenic for the MHC-II GP_61−80_ LCMV epitope, thus the antigen presentation is not directly proteasome dependent) with ONX 0914 or vehicle 2 h before i.v.-infection with LCMV. Almost all splenic CD4+ T cells were activated within 18 h after LCMV infection as measured by CD69 up-regulation (Figure 2A). CD25 was up-regulated to a lesser extent than CD69 (Figure 2B). Importantly, both CD69 and CD25 were ~45% less up-regulated on T cells from ONX 0914-treated mice, corroborating attenuated T cell activation *in vivo*.

### ONX 0914-treatment did not impair NF-κB signaling, but reduced ERK-phosphorylation sustainment

**Figure 3:**
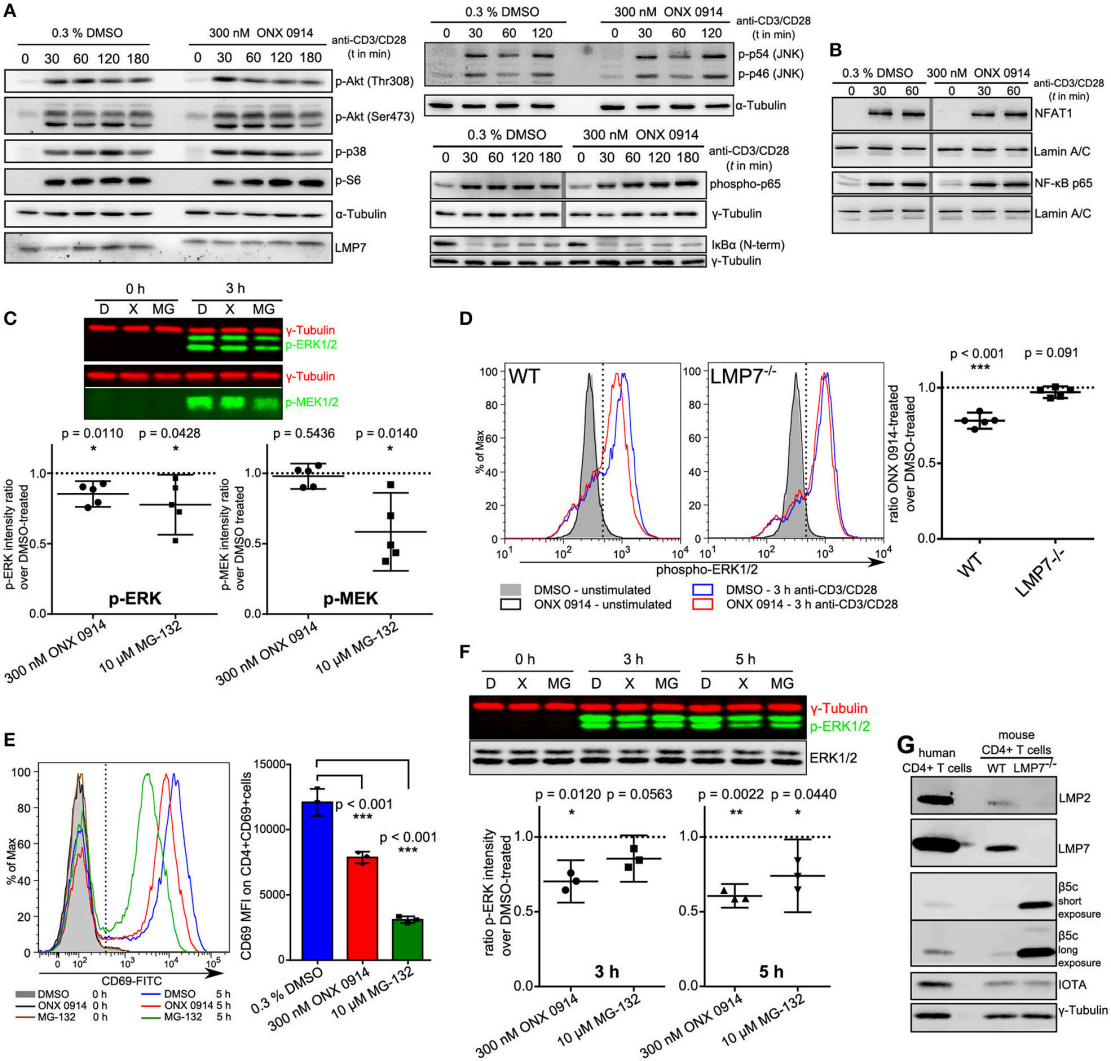
ONX 0914 reduces ERK-phosphorylation sustainment while leaving most canonical signaling pathways unaffected. **(A)** Immunoblot analysis with whole cell lysates for indicated (phospho-)proteins in expanded CD4+ T cells after pulse-treatment with ONX 0914 or DMSO for 2 h, followed by activation with plate-bound anti-CD3/anti-CD28 antibodies for indicated time periods. Representative blots of three independent experiments are shown. Alpha-tubulin was used as control. Vertical gray line indicates signals from the same membrane and detection, but not originally juxtaposed. **(B)** Immunoblots of p65 and NFAT with nuclear fractions of cell lysates after activation of cells treated as in **A**. Lamin was used as a loading control. Vertical gray line indicates signals from same membrane, but not originally juxtaposed. **(C)** Cells treated as in A were lysed and used for near-IR immunoblotting against indicated proteins (top: representative example). Intensities of p-ERK and p-MEK relative to tubulin loading control at 3 h were quantified in five independent experiments. Graphs below show mean + 95% CI. Ratios of ONX 0914-treated/DMSO-treated signals were analyzed with one-sample *t*-test, μ_0_ = 1, *p*-values as indicated in the figure. **(D)** MACS-enriched splenic CD4+ T cells from WT or LMP7^−/−^ mice were 2 h pulse-treated with DMSO or ONX 0914 and activated for 3 h with plate-bound anti-CD3/anti-CD28 antibodies or left unstimulated. Intracellular p-ERK1/2 levels were measured using flow cytometry on CD4+ p-ERK+ cells. Histograms (left) show one representative example of five independent experiments. Phospho-ERK+ median fluorescence intensity ratios of ONX 0914-treated/DMSO-treated cells from five independent experiments are shown as mean + 95% CI in the right hand graphs. One-sample *t*-test with μ_0_ = 1, *p*-values as indicated. **(E)** MACS-enriched human CD4+ T cells were pulse-treated with DMSO or ONX 0914 or continuously treated with MG-132 and activated with stimulating beads for 5 h. CD69 expression on CD4+ CD69+ cells was measured by flow cytometry and median fluorescence intensities used for quantification. Representative histogram (left) and quantification with pooled data of three independent experiments (right) are shown (mean ± SD); two-way repeated measures ANOVA, Sidak's post-test, *p*-values as indicated. **(F)** Cells treated as in **E** were lysed and used for near-IR detection of p-ERK1/2 intensities normalized to γ-tubulin after 3 and 5 h of activation. Top: Representative immunoblot, graph below: Ratio of ONX 0914-treated/DMSO-treated signals from three independent experiments (mean with 95% CI) was analyzed with one-sample *t*-test, μ_0_ = 1, *p*-values are indicated. **(G)** Immunoblot analysis of murine CD4+ T cells from WT or LMP7^−/−^ mice compared with purified human CD4+ T cells. One example out of three similar experiments is presented.

To investigate whether canonical TCR/CD28 signaling was altered by ONX 0914 treatment we used primary mouse T cells after PMA/ionomycin stimulation and 1 week of culture in IL-2 containing medium (37). Within 7 days the cells reached a resting state, had similar LMP7 and β5c expression compared with naïve cells (Figure S1B in Supplementary Material, referred to as “expanded T cells”) and re-up-regulated CD69 upon TCR-triggered re-stimulation, which was reduced by ONX 0914-treatment (Figure S1C in Supplementary Material). Therefore, we used expanded T cells to investigate into signaling pathways by immunoblotting. Within the first 3 h of activation, most analyzed signaling pathways appeared unaffected by ONX 0914, including phosphorylation of p38, JNK, Akt(Thr308), Akt(Ser473), S6 ribosomal protein (Figure 3A), and nuclear translocation of NFAT (Figure 3B). Given that the proteasome is a well-known regulator of NF-κB signaling (38–40), we carefully analyzed not only degradation of IκBα (Figure 3A, Figures S2C–E), but also p65-phosphorylation and p65 nuclear translocation (Figures 3A,B). All analyzed NF-κB signaling components remained unaffected by ONX 0914 in both expanded and naïve T cells, in line with previous results (15, 22). However, we noticed a trend toward reduced ERK1/2 phosphorylation 3 h after activation in WT, but not in LMP7-deficient cells, in enhanced-chemiluminescence-based immunoblots (Figure S2A in Supplementary Material). We then quantified ERK-phosphorylation using near-infrared-fluorescent-dye-labeled antibodies and compared it to the phosphorylation of its upstream kinase MEK. Strikingly, while ONX 0914-treatment did not affect MEK-phosphorylation, it did reduce ERK-phosphorylation by 14.8% (±7.4%) in expanded T cells, while total proteasome inhibition with MG-132 reduced both, MEK-phosphorylation and ERK-phosphorylation (Figure 3C). As ERK-signaling is central to both CD69 and IL-2 expression (41, 42) and underlies a digital signaling modality in lymphocytes (43) we aimed to corroborate the results using intracellular flow cytometry measuring ERK-phosphorylation directly in naïve T cells at single-cell resolution. Our data revealed that ONX 0914-treatment significantly reduced ERK-phosphorylation by 21.9% (±4.3%) in naïve T cells from WT, but not LMP7-deficient mice (3.1% ± 3.1%, Figure 3D). In contrast, total ERK remained unaffected (Figure S2B in Supplementary Material). Additionally, ONX 0914 and MG-132 also reduced CD69 up-regulation (Figure 3E) and ERK-phosphorylation (Figure 3F) in primary human CD4+ T cells, which also expressed β5c at a low and LMP7 at a high level (Figure 3G). These results validated that ONX 0914-treatment selectively reduced TCR-induced ERK-phosphorylation sustainment in primary T cells.

### IP inhibition induced a mild proteostasis-stress in activated T cells

**Figure 4:**
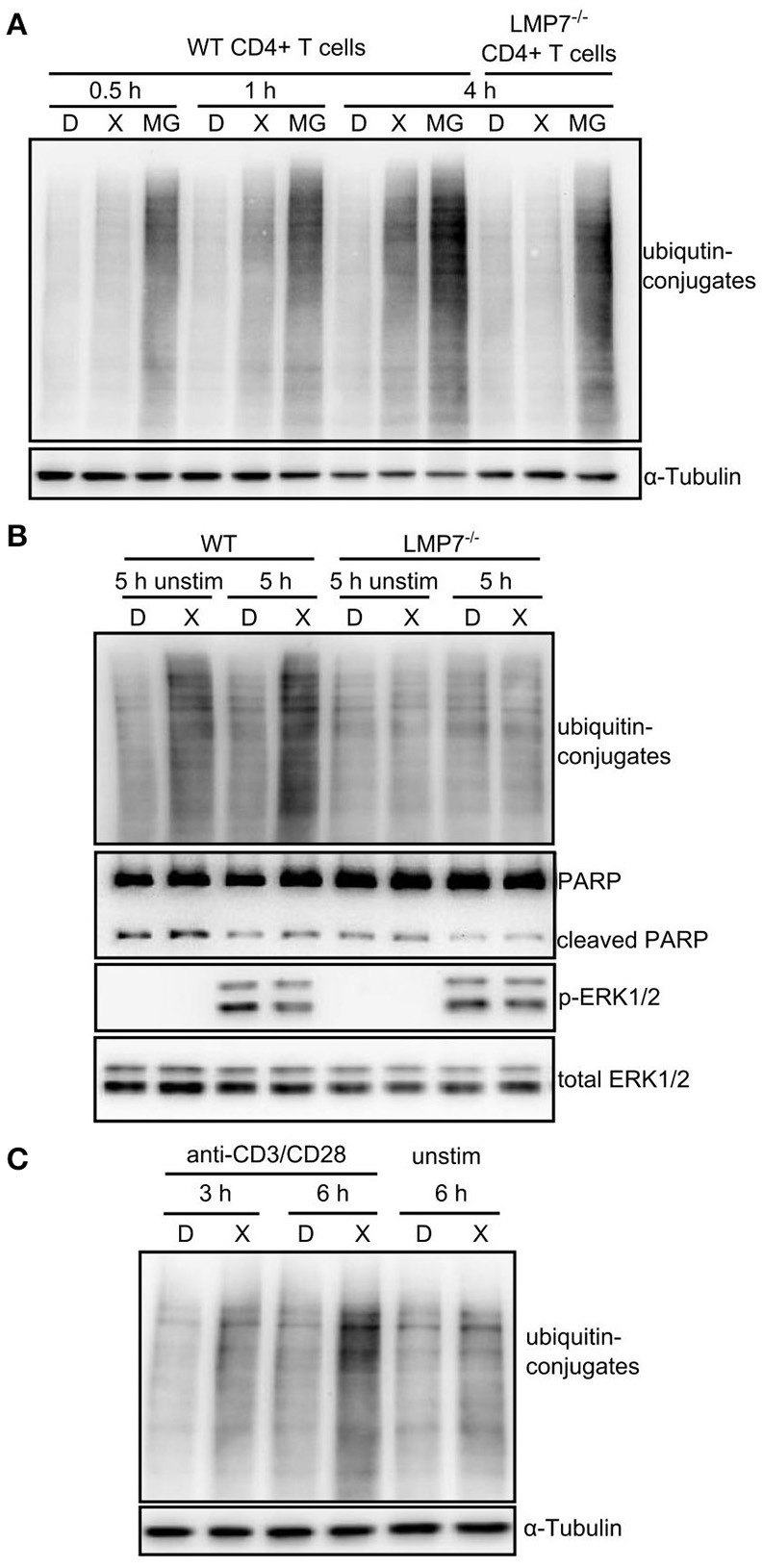
Immunoproteasome inhibition induces mild proteostasis stress in activated CD4+ T cells. **(A)** Expanded CD4+ T cells from WT or LMP7-deficient mice were pulse-treated for 2 h with 0.3% DMSO (D) or 300 nM ONX 0914 (X) or continuously treated with 10 μM MG-132 (MG) and consequently activated with plate-bound anti-CD3/anti-CD28 antibodies for the indicated time periods. Immunoblot analysis of ubiquitin conjugates with α-tubulin as loading control is shown. A representative example of at least three experiments with similar outcome is displayed. **(B)** Primary MACS-enriched naive CD4+ T cells from WT or LMP7-deficient mice were pulse-treated for 2 h with 0.3% DMSO (D) or 300 nM ONX 0914 (X) and activated with plate-bound anti-CD3/anti-CD28 antibodies or left unstimulated for 5 h. Immunoblot analyses against indicated proteins with total ERK as loading control are displayed. One example of at least three experiments with similar outcome is presented. **(C)** Expanded CD4+ T cells from WT mice were pulse-treated for 2 h with 0.3% DMSO (D) or 300 nM ONX 0914 (X) and activated with plate-bound anti-CD3/anti-CD28 antibodies for indicated time periods or were left unstimulated. Immunoblot analysis for ubiquitin-conjugates with α-tubulin serving as loading control. One example of at least three experiments with similar outcome is shown.

ONX 0914-treatment did not induce ubiquitin-conjugate accumulation in the T cell line Molt4 as reproduced here (Figure S3A in Supplementary Material) (22). However, we tested whether IP inhibition induced ubiquitin-conjugate accumulation in primary T cells. We pulse-treated expanded CD4+ T cells from WT and LMP7-deficient mice with DMSO or ONX 0914 before activation and compared them to cells that were continuously treated with MG-132. While MG-132-treated cells immediately showed ubiquitin-conjugate accumulation, ONX 0914-treated cells did not show such effects at early time points, resembling previous data. Nevertheless, ONX 0914-treatment induced a robust accumulation of ubiquitin-conjugates after 3–4 h in WT, but not LMP7-deficient cells (Figure 4A). Notably, when cells were left unstimulated, less ubiquitin-conjugates accumulated (Figures 4B,C) indicating that the bulk of proteostasis-stress was activation-induced and not due to steady-state proteostasis.

### IP inhibition reduced dual specificity phosphatase (DUSP)5 and enhanced DUSP6 protein levels during T cell activation

**Figure 5:**
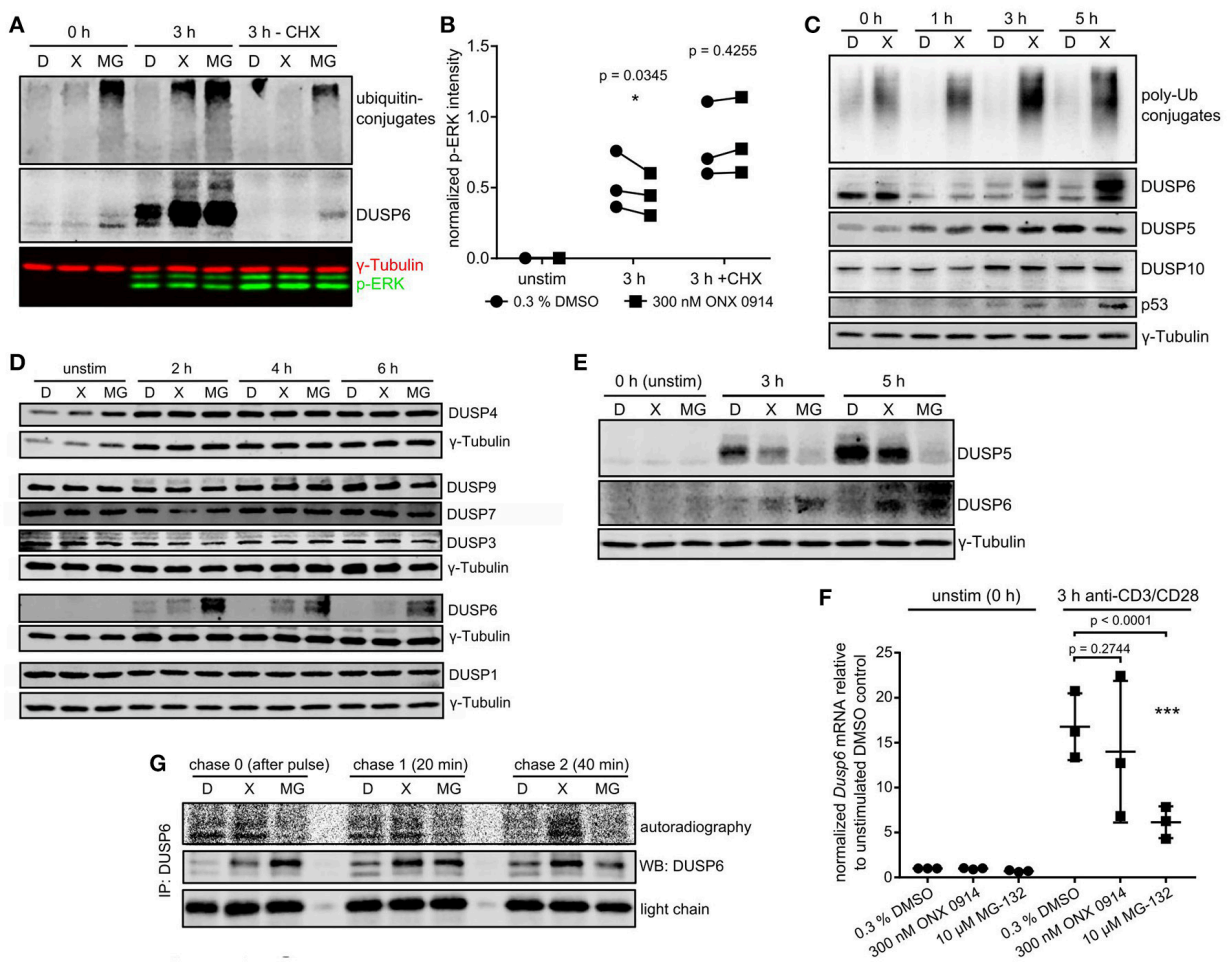
Immunoproteasome inhibition dysregulates DUSP5 and DUSP6 in T cells. **(A)** Expanded murine CD4+ T cells were pulse treated with 0.3% DMSO (D) or 300 nM ONX 0914 (X) or continuously treated with 10 μM MG-132 (MG) before activation with plate-bound anti-CD3/anti-CD28 antibodies for 3 h in the presence or absence of cycloheximide (CHX). Shown are immunoblots for ubiquitin and DUSP6 employing γ-tubulin as loading control. One example of three independent experiments is shown. **(B)** Quantification of p-ERK intensities normalized to γ-tubulin from three independent experiments performed as in **A**. Paired *t*-test, *p*-values as indicated. **(C)** MACS-enriched naive murine CD4+ T cells from WT mice were pulse-treated with 0.3% DMSO (D) or 300 nM ONX 0914 (X) for 2 h before activation with anti-CD3/anti-CD28 antibodies. Cells were lysed after indicated times and immunoblots performed as indicated. One example of at least three similar experiments. **(D)** Expanded murine CD4+ T cells were pulse-treated with 0.3% DMSO (D) or 300 nM ONX 0914 (X) or continuously treated with 10 μM MG-132 (MG) before activation with plate-bound anti-CD3/anti-CD28 antibodies for indicated time periods. Immunoblots were performed with indicated antibodies. One example of three or more independent reproductions is shown. **(E)** One example of three independent reproductions of the experiment as in **C**, but with primary human CD4+ T cells from healthy donors and continuous MG-132 treatment (10 μM) in addition. **(F)** Expanded murine CD4+ T cells treated as in **D** were used for RNA extraction and q-RT-PCR against *Dusp6* after 3 h of activation. Fold change of *Dusp6* mRNA (normalized to *Rpl13* and *Ipo8*) over unstimulated DMSO control. Pooled data from three independent experiments. Two way ANOVA, Sidak's post test, *p*-values as indicated. **(G)** T1 cells were treated with 200 U/ml IFN-γ for 3 days to induce higher immunoproteasome content. Cells were then pulse-treated for 2 h with DMSO, ONX 0914, or MG-132 as in **C**. Consequently, cells were activated with plate-bound anti-CD3/CD28 antibodies in RPMI 1640 +supplements. After 1–2 h the cells were starved in methionine/cysteine-free RPMI 1640 for 1 h, followed by a 15 min radioactive pulse of ^35^S-cys/met in RPMI 1640 at 250 μCi/ml. Cells were washed and lysed directly after (chase 0) as well as 20 and 40 min after the pulse. Lysates were loaded for an anti-DUSP6-IP according to β-count CPM. After 6 h of IP and washing, the radioactive signal of newly synthesized DUSP6 was detected with a phosphoimager. Total DUSP6 in the IP and the antibody light chain were used as loading controls. One example of three experiments with similar outcome is displayed.

We wondered whether a link between proteostasis stress and reduced ERK-phosphorylation existed. Treatment with the protein synthesis inhibitor cycloheximide abolished ubiquitin-conjugate accumulation in ONX 0914-treated cells, but not in MG-132-treated cells (Figure 5A), supporting that protein neo-synthesis was the primary driver of proteostasis stress after IP inhibition. Importantly, cycloheximide-treatment also enhanced overall ERK-phosphorylation and abolished the relative reduction in ERK-phosphorylation after ONX 0914-treatment (Figures 5A,B). This suggested that a *de novo* synthesized feedback regulator or a ubiquitin-conjugate-depending mechanism mediated impaired ERK-phosphorylation. We therefore hypothesized an involvement of dual specificity phosphatases. Particular DUSPs were shown (i) to be constitutively expressed or activation-induced in T cells, (ii) to specifically target individual substrates like ERK, and (iii) to be regulated via phosphorylation, ubiquitination, and proteasomal degradation (44, 45). We tested candidate DUSPs in primary T cells (Figures 5C,D) and in the T1 cell line (Figure S4E). T1 cells pre-treated with IFN-γ expressed only LMP7 containing, but no β5c containing proteasomes (Figure S4C in Supplementary Material) and showed several similar effects of ONX 0914-treatment as observed in primary T cells (Figures S4A,B). While most analyzed DUSPs remained unaltered by ONX 0914 (Figures 5C,D, Figure S4E) we found that DUSP5 was reduced after 5 h (Figure 5C, Figures S3B, S4E in Supplementary Material). In contrast, the ERK-specific DUSP6 accumulated within 3–5 h in murine and human T cells treated with ONX 0914 or MG-132 (Figures 5A,C–E). Importantly, naïve or expanded LMP7-deficient cells did not show these effects with or without ONX 0914 (Figures S3B,C in Supplementary Material). The increased DUSP6 protein levels in ONX 0914-treated cells were due to impaired degradation of DUSP6 as radioactively pulse-labeled DUSP6 accumulated, but *Dusp6* mRNA-levels were not enhanced (Figures 5F,G). In MG-132-treated cells also steady-state DUSP6 accumulated in cycloheximide-treated cells, which was not found after ONX 0914-treatment (Figure 5A). Congruent with reducing mRNA levels, MG-132-treatment also impaired radioactive ^35^S-incorporation into DUSP6 protein (Figures 5F,G, Figure S4D in Supplementary Material). Together, these results confirmed that DUSP6 had fast turnover kinetics (46) in control cells, while ONX 0914-treatment impaired only DUSP6 degradation and MG-132-treatment fully blocked DUSP6 degradation, but also impaired *Dusp6* expression. In line with this notion, we observed that DUSP6 was expressed at basal state in naïve T cells, but was initially degraded after T cell activation in both DMSO and ONX 0914 treated cells (Figure 5C). Only after prolonged activation (3 and 5 h) DUSP6 accumulated relative to DMSO treated cells (Figure 5C) showing that ONX 0914 hindered the rapid degradation of *de novo* synthesized DUSP6, but did not block its initial degradation. Thus, the degree of proteasome inhibition as induced by subunit-selective targeting in contrast to broad proteasome inhibition determined the impact on a proteasome regulated process in T cells.

### Effects of ONX 0914 on CD69, IL-2, and ERK-signaling were not caused by DUSP6 accumulation alone

DUSP6 was characterized as a regulator of ERK-signaling during activation of mouse and human T cells (47–52). Furthermore, phosphorylated, degradation-primed DUSP6 retained its phosphatase activity (53). Hence, our data strongly suggested a functional involvement of DUSP6 in the effect of ONX 0914 on ERK either via direct de-phosphorylation or via cytosolic retention (54). We used confocal microscopy to assess nuclear translocation of total and phosphorylated ERK in activated T cells after IP inhibition. Nuclear intensities of both p-ERK and total ERK increased after activation. Reduced ERK-phosphorylation was detectable in the nucleus after ONX 0914 and MG-132-treatment, while total nuclear translocation of ERK was unaffected (Figures S5A,B in Supplementary Material) indicating that the effect on ERK-phosphorylation might be mediated by a nuclear phosphatase rather than the cytosolic DUSP6. To clarify if DUSP6 played a functional role, we used primary T cells from littermate DUSP6^−/−^, DUSP6^+/−^, and age-/sex-matched DUSP6^+/+^ mice. DUSP6^−/−^ cells up-regulated CD69 and secreted IL-2 to the same extent as WT cells and were equally affected by ONX 0914-treatment (Figures S6A–D in Supplementary Material). Furthermore, ERK-phosphorylation at 3 h after activation was unaltered between T cells from WT, DUSP6-heterozygous or DUSP6-deficient mice and the reduction of ERK-phosphorylation by ONX 0914 was comparable between the groups (Figures S6E,F in Supplementary Material). Importantly, DUSP6-heterozygosity and DUSP6-deficiency did not alter LMP7- or β5c-content (Figure S6G in Supplementary Material). Taken together, our data did not support a non-redundant involvement of DUSP6 in the ONX 0914-mediated effects on T cell activation, disconfirming a direct causative relationship between DUSP6 accumulation and impaired T cell activation.

### T cells alleviate IP-inhibition-mediated proteostasis stress without apoptosis induction

**Figure 6:**
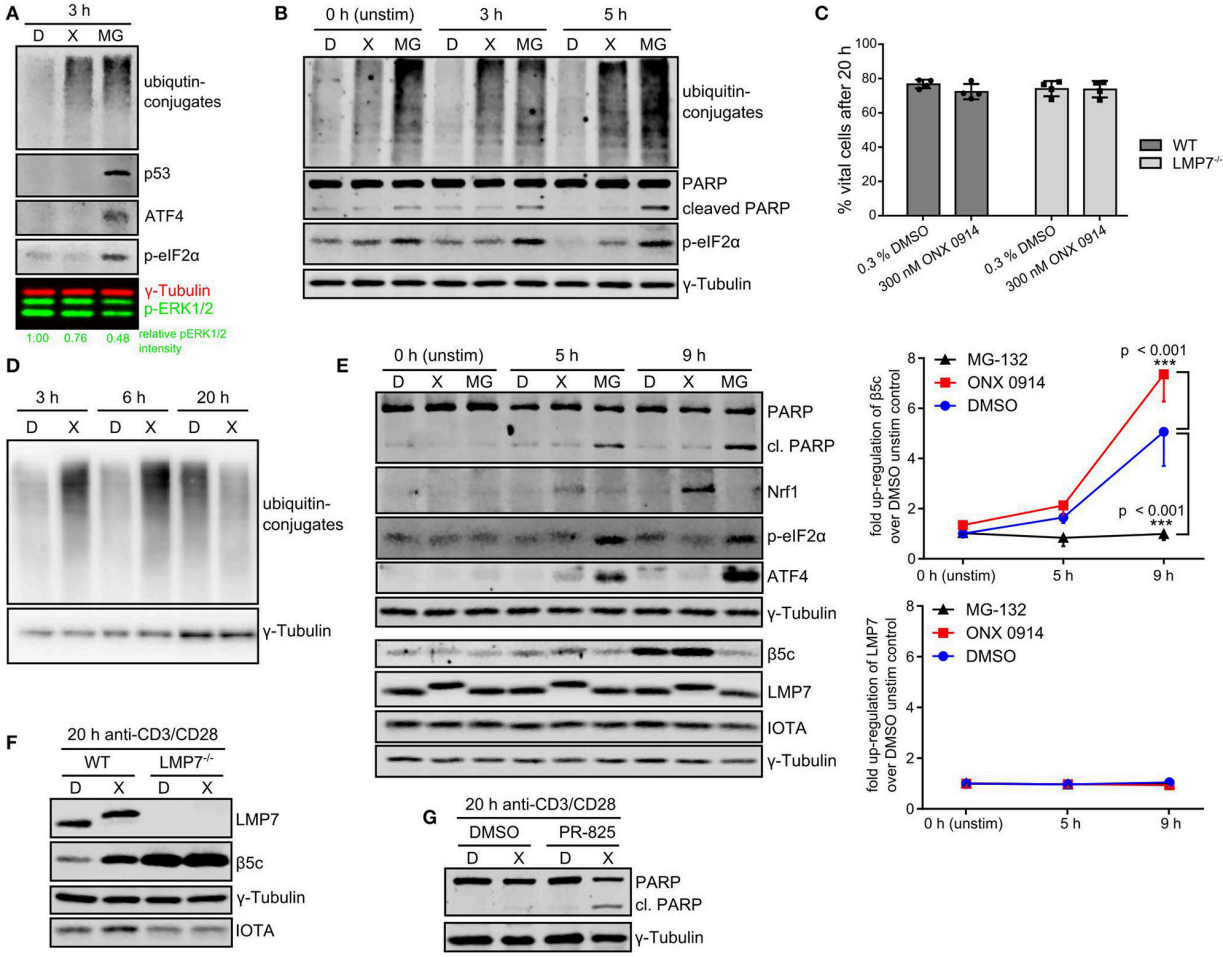
T cells alleviate IP-inhibition-induced proteostasis stress without induction of apoptosis. **(A)** Expanded murine CD4+ T cells were pulse treated with 0.3% DMSO (D) or 300 nM ONX 0914 (X) or continuously treated with 10 μM MG-132 (MG) before activation with plate-bound anti-CD3/anti-CD28 antibodies for 3 h. Immunoblot analysis of indicated proteins with γ-tubulin as loading control and phosphorylated ERK1/2 as activation control was performed. Numbers below show quantification of normalized p-ERK as in Figure 3C. One example of three independent experiments is displayed. **(B)** Primary human CD4+ T cells purified from PBMCs of voluntary healthy donors were pre-treated as in A and stimulated with antibody-coated beads against CD3/CD28/CD2 (Miltenyi human T cell activation kit) for indicated time periods. Immunoblot analysis was performed for indicated proteins. γ-Tublin served as loading control. One example out of three independent experiments is shown. **(C)** Naive T cells isolated from either WT or LMP7-deficient mice were pulse-treated for 2 h with 0.3% DMSO (D) or 300 nM ONX 0914 (X) and activated with plate-bound antibodies against CD3/CD28 for 20 h. The percentage of vital cells was assessed using AO/PI staining in a Cellometer 2000. Pooled data from four independent experiments is displayed. **(D)** Cells treated as in **C** were harvested after 3, 6, or 20 h. Immunoblot analysis against ubiquitin-conjugates is shown with γ-tubulin as loading control. One example of at least three experiments with similar outcome is shown. **(E)** Cells treated as in **C** (with continuous MG-132-treatment in addition) were lysed after indicated time periods. Immunoblots against indicated proteins with γ-tubulin as loading control are shown. One example out of three independent experiments is shown. Quantification of normalized β5c and LMP7 intensities from three independent experiments is shown in the right hand graph (mean + SD for each time point). **(F)** Cells treated as in **C** were harvested after 20 h. Immunoblot analysis against indicated proteins with γ-tubulin as loading control and IOTA as total proteasome control is presented. One representative example of at least three independent experiments is shown. **(G)** WT CD4+ T cells treated as in **C** were activated and PR-825 was added to 100 nM final concentration after 4 h. Cells were harvested 20 h after activation and immunoblots performed as indicated. One example out of three experiments is shown.

The observed ubiquitin-conjugate accumulation prompted us to study the consequence for cell function and survival. As expected, MG-132-treatment induced stress response pathways like p53 accumulation, ATF4 induction and eIF2α-phosphorylation (Figure 6A). Interestingly, ONX 0914-treatment did not induce these pathways in expanded T cells nor did it induce enhanced apoptosis compared to DMSO-treatment in murine or human T cells as analyzed by PARP cleavage (Figures 6B,E, Figures S3B,C). Naïve T cells showed some p53 accumulation within the first hours after ONX 0914-treatment (Figure 5C), but retained viability even 20 h after activation (Figure 6C). On the contrary, primary activated T cells could alleviate the amount of ubiquitin-conjugates within 20 h (Figure 6D). This was not attributed to LMP7 up-regulation as all detectable LMP7 remained electrophoretically shifted by covalent modification with ONX 0914 even 20 h after pulse-treatment (Figures 6E,F). In contrast, we detected increased β5c protein levels, indicating that proteostasis stress might be alleviated via standard proteasome up-regulation (Figure 6F). To assess whether the well-documented Nrf1-pathway might be involved (55–57), we analyzed β5c and Nrf1 protein levels 5–9 h after activation. Both, DMSO and ONX 0914-treated cells up-regulated β5c, but we also detected soluble Nrf1 in lysates from ONX 0914-treated cells correlating with slightly increased β5c protein levels (Figure 6E). No soluble Nrf1 was detected in MG-132-treated cells, likely due to aggregation and Nrf1-insolubility (58–60). Also, β5c up-regulation was abrogated in MG-132-treated cells (Figure 6E). Next, we pre-treated naïve T cells with ONX 0914 or DMSO and added the β5c inhibitor PR-825 after the first 4 h of activation. Co-inhibition of β5c resulted in enhanced PARP-cleavage in ONX 0914-treated cells after 20 h corroborating that β5c up-regulation contributed to preserving viability (Figure 6G). ONX 0914-treatment similarly affected B cell CD69-up-regulation, integrated-stress response as well as β5c and Nrf1, but B cells showed enhanced PARP-cleavage also after ONX 0914-treatment indicating higher susceptibility to apoptosis (Figures S7A–D in Supplementary Material). Our data thus reveal that IP inhibition-induced mild protein stress was partially or fully alleviated by primary lymphocytes.

## Discussion

IP inhibition with ONX 0914 showed efficacy for the treatment of autoimmune pathologies in several pre-clinical models and impaired T cell polarization and cytokine secretion (14, 20, 22, 24, 25). KZR-616, structurally closely related to ONX 0914, is now tested in clinical trial (clinical trial ID: NCT03393013). Studies indicated that altered T cell functions are caused by T cell-intrinsic action of ONX 0914 (14, 20, 61), but it remained elusive how this is explained mechanistically. In a previous study, we found that both targeting β5c in LMP7^−/−^ mice or LMP7 in WT mice can ameliorate symptoms in experimental autoimmune encephalomyelitis in a bone-marrow-cell-dependent manner, rendering reduced proteasome activity rather than a strictly LMP7-dependent factor as the likely underlying mechanism (20). Here, we report novel mechanistic insight into the effects of ONX 0914 in primary murine and human lymphocytes. We find that ONX 0914 impaired lymphocyte activation within the first hours, which was accompanied by reduced ERK-phosphorylation while NF-κB-signaling was unaffected. Beling and colleagues recently also reported a minor effect on ERK-phosphorylation by ONX 0914 in mouse macrophages (15, 30) and unaffected NF-κB-signaling in spite of IP inhibition was also reported previously (15, 22). Our data support the hypothesis that dysregulated phosphatases might be involved as the upstream-kinase MEK was unaffected and at least two phosphatases are affected by IP inhibition. Importantly, the effects obtained with ONX 0914 differ significantly from broad-spectrum proteasome inhibition with MG-132, which also impaired *Dusp6* expression, while ONX 0914 mainly impaired DUSP6 degradation without fully blocking it. Experiments with DUSP6^−/−^ T cells show that DUSP6 is dispensable for the observed effects of ONX 0914 on T cell activation. We observed no enhanced ERK-phosphorylation in DUSP6-deficient cells, which is in conflict with previous studies (47, 50, 52). We thus disconfirm the hypothesis that DUSP6 alone is responsible for impaired ERK signaling, but DUSP6 might influence other processes not investigated here and compensation by other phosphatases might occur as high redundancy is a reported hallmark of DUSPs (44, 45). In contrast, reduced DUSP5 expression is more likely a downstream effect of reduced ERK-signaling (62, 63) and the relevance of this for impaired T cell activation remains to be investigated. Our microscopy analysis suggests the involvement of a nuclear phosphatase, but DUSP1 and DUSP4 were unaffected by proteasome inhibition, while we were unable to detect DUSP2 and DUSP16. Further investigation is required to identify the responsible mechanism for reduced ERK-signaling.

Apart from altered signaling, we also found that IP inhibition induced mild proteostasis stress in primary lymphocytes. Originally, ONX 0914 was not found to result in ubiquitin-conjugate formation in a T cell-derived cell line, as reproduced here (22). Therefore, partial inhibition of IPs was considered unlikely to have a major impact on total proteostasis. Two new findings have changed our knowledge in this respect. First, recent evidence unraveled that pre-clinically efficacious doses of ONX 0914 inhibit both, LMP7 and LMP2 (64), which explains the LMP2-co-dependency of ONX 0914-treatment effects in T cell activation (Figure 1). Second, while IP expression in lymphoid cells is known for decades, the high extent to which IPs account for the total proteasome content of naïve lymphocytes was underestimated. We find that T and B cells contain almost only LMP7-containing proteasomes already at the naïve state while LMP7-deficient cells contain almost exclusively standard proteasomes in line with reported incorporation interdependency (8, 65). Furthermore, activated T cells undergo metabolic and proteomic re-programming (66, 67) which likely demands high proteasome capacity as also indicated by slightly increased ubiquitin-conjugates after 20 h in DMSO-treated cells (Figure 6D). Hence, high IP content and activation-induced metabolic demands render primary lymphocytes highly susceptible to proteostasis stress after IP inhibition. Indeed, we find impaired ubiquitin-conjugate clearance after activation in both human and murine cells. Interestingly, T cells retained viability, while B cells were more susceptible to apoptosis induction. This might explain why ONX 0914 showed high efficacy in ameliorating lupus-like symptoms (21) and in preventing chronic rejection after kidney transplantation (26) as plasma cells are likely even more affected by reduced proteasome capacity as compared to naïve B cells. T cells were instead able to clear ubiquitin-conjugates after ONX 0914-treatment with prolonged activation. Unexpectedly, this alleviation was not due to activation-induced formation of new unmodified IPs as we observed no LMP7 neo-synthesis in response to ONX 0914-treatment after activation (Figure 6). In contrast, T cell activation induced β5c up-regulation, but not after MG-132-treatment, possibly because of p-eIF2α-mediated synthesis-inhibition. Notably, β5c up-regulation was transient, as expanded T cells had comparable LMP7 and β5c content to naïve T cells (Figure S1). ONX 0914-treatment boosted β5c up-regulation (likely via Nrf1) which contributed to retaining cell viability (Figure 6). Our data are hence in contrast to the increase of LMP7 after T cell activation reported by Sula Karreci et al. who also report impaired proliferation of T cells with the novel LMP7-inhibitor DPLG3, which did not detectably inhibit LMP2 even at high concentrations *in vitro* (27). Thus, early and prolonged activation might be differently affected by dual or single subunit inhibition. We have only characterized the proteasome subunit content in naïve CD4+ T cells and naïve B cells. With respect to therapeutic efficacy *in vivo*, however, it would be of interest to determine the proteasome subunit content in relevant T cell subpopulations including Th1, Th2, Th17, and regulatory T cells as well as effector and memory T cells of both the CD4+ and the CD8+ compartment. Different proteasome subunit compositions of individual subsets, but also different demands for proteasome capacity, might have an impact on how immunoproteasome inhibition influences T cell responses *in vivo* and in different pathological settings. These aspects will have to be addressed in future studies. Our data also show that essentially IPs are dispensable for T cell activation and can be fully substituted by standard proteasomes as LMP7-deficient cells behaved like untreated WT cells. Together, our data are thus in clear contrast to the concept that LMP7-containing proteasomes are superior over standard proteasomes in clearing ubiquitin-conjugates as reported by Seifert et al. (16), which was challenged by other studies before (17, 18). Why naïve lymphocytes have high IP content remains elusive. Because of the high constitutive expression of IPs in lymphocytes, we propose to omit the phrasing “constitutive proteasome,” but instead use the phrasing “standard proteasome” as opposed to immunoproteasome. As immunoproteasomes are expressed in immune cells and at sites of inflammation, less adverse effects of immunoproteasome inhibitors as compared to broad-spectrum proteasome inhibitors should be expected in clinical application. Indeed, long-term treatment with ONX 0914 in mice and rats has shown no obvious adverse effects (21, 26, 29). Furthermore, ONX 0914-treated mice can well control viral infections (22, 30, 68, 69). However, we have observed that ONX 0914-treatment caused enhanced susceptibility to experimental intravenous infection with *Candida albicans*, but additional fungicidal drug treatment fully suppressed *Candida* growth in the presence of ONX 0914 (70). The structural relative of ONX 0914, KZR-616, designed for selective inhibition of LMP7 and LMP2, was well-tolerated by healthy volunteers in phase I clinical trial (71). Hence, immunoproteasome inhibition appears to be a safe approach for future treatment of inflammatory diseases in humans. In summary, our study describes impaired proteostasis during lymphocyte activation as the underlying molecular mechanism of ONX 0914-treatment, which is attributed to both IP content and activation state of the cell. This provides a mechanistic rationale why IPs are such promising treatment targets in a wide range of immunopathologies with less negative side effects to be expected as compared to approved proteasome inhibitors today.

## Author contributions

CS planned, performed and analyzed the majority of experiments, and wrote the manuscript. TB performed and analyzed experiments. MG designed and co-supervised the project, provided funding, and revised the manuscript. MB designed and supervised the project, performed experiments, provided funding, and revised the manuscript.

### Conflict of interest statement

The authors declare that the research was conducted in the absence of any commercial or financial relationships that could be construed as a potential conflict of interest.

## Supplementary material

Presentation 1 Macro nuclear intensity measurement.

Data Sheet 1 Supplementary methods and figures.
