# Supplementary material for: Immunoproteasome Inhibition Impairs T and B Cell Activation by Restraining ERK Signaling and Proteostasis
Source: Front Immunol. 2018 Oct 26;9:2386. doi: 10.3389/fimmu.2018.02386 (PMC6212513; doi:10.3389/fimmu.2018.02386)
Supplement: Data Sheet 1 — Supplementary methods and figures. [file Data_Sheet_1.pdf]

Supplementary Material

Immunoproteasome inhibition impairs T and B cell activation by restraining ERK signaling and proteostasis

Christian Schmidt[1,2], Thilo Berger[1], Marcus Groettrup[1,2,3], Michael Basler[1,2,3*]

[1]Chair of Immunology, Department of Biology, University of Konstanz, Universitätsstraße 10, D-78464 Konstanz, Germany

[2]Konstanz Research School Chemical Biology (KoRS-CB), University of Konstanz, D-78457 Konstanz, Germany

[3]Biotechnology Institute Thurgau at the University of Konstanz (BITg), Unterseestr. 47, CH-8280 Kreuzlingen, Switzerland

*** Correspondence:**
Michael Basler, University of Konstanz, Chair of Immunology, Universitätsstraße 10, D-78464 Konstanz, Germany , phone: +49 7531 882258, e-mail: Michael.Basler@uni-konstanz.de

List of Reagents and Resources:

REAGENT or RESOURCE	SOURCE	IDENTIFIER
Antibodies		
anti-p-ERK1/2	Cell Signaling	Cat#4370
anti-ERK1/2	Cell Signaling	Cat#4695
anti-p-JNK	Cell Signaling	Cat#9255
anti-p-Akt(Ser473)	Cell Signaling	Cat#4060
anti-p-Akt(Thr308)	Cell Signaling	Cat#13038
anti-NFAT1	Cell Signaling	Cat#5861
anti-phospho-p65	Cell Signaling	Cat#3033
anti-NF-κB p65	Cell Signaling	Cat#8242
anti-p-MEK1/2	Cell Signaling	Cat#2338
anti-p-eIF2α	Cell Signaling	Cat#3398
anti-ATF4	Cell Signaling	Cat#11815
anti-p53	Cell Signaling	Cat#2524
anti-PSMB5 (β5c)	Cell Signaling	Cat#12919
anti-PSMB6 (β1c)	Cell Signaling	Cat#13267
anti-PSMB7 (β2c)	Cell Signaling	Cat#13207
anti-Lamin A/C	Cell Signaling	Cat#2032
anti-DUSP10	Cell Signaling	Cat#3483
anti-phospho-p38	Cell Signaling	Cat#9215
anti-p-S6	Cell Signaling	Cat#4858
anti-p-IκBα	Cell Signaling	Cat#2859

anti-IκBα (N-term)	Cell Signaling	Cat#4814
anti-PARP	Cell Signaling	Cat#9532
anti-CHOP	Cell Signaling	Cat#2895
anti-TCF11/Nrf1	Cell Signaling	Cat#8052
anti-DUSP16	Cell Signaling	Cat#5523
anti-DUSP3 (VHR)	Santa Cruz Biotechnology	Cat#sc-274161
anti-DUSP9 (MKP-4)	Santa Cruz Biotechnology	Cat#sc-377106
anti-DUSP7 (PYST-2)	Santa Cruz Biotechnology	Cat#sc-137010
Mono-and-polyubiquitinated conjugates monoclonal Ab (FK2)	ENZO Lifesciences	Cat#BML-PW8810
anti-DUSP4 (MKP-2)	BD Biosciences	Cat#610850
anti-DUSP6 (MKP-3)	Abcam	Cat#ab220811
anti-DUSP5	Abcam	Cat#ab200708
anti-γ-Tubulin	Sigma-Aldrich	Cat#T6557
anti-α-Tubulin	Sigma-Aldrich	Cat#T5168
anti-LMP7 (mouse)	Polyclonal rabbit antiserum, in house	Khan 2001 (1)
anti-LMP2 and anti-MECL-1	Polyclonal rabbit antiserum, (provided by Benoit J Van den Eynde, Ludwig Cancer Research, Brussels Branch, Brussels, Belgium)	Guillaume 2010 (2)
anti-IOTA	Monoclonal, IB5, obtained from Klaus Scherrer (Institute Jaques Monod, Paris, France)	Kremer 2010 (3)
anti-LMP7 (human)	Polyclonal rabbit antiserum, in house	Macagno 1999 (4)
anti-mouse CD69-FITC	ebioscience	Cat#11-0691-85
anti-human CD69-FITC	Biolegend	Cat#310904
anti-mouse CD4-APC	ebioscience	Cat#17-0041-83
anti-human CD4-APC	BD Pharmingen	Cat#555349
anti-mouse CD25-PE	Biolegend	Cat#101904
anti-mouse CD19-APC	Biolegend	Cat#115512
Anti-rabbit Alexa647 F(ab')2 fragment	Invitrogen	Cat#A21246
anti-CD3 clone 17A2	Biologend	Cat#100202
anti-CD28 clone 37.51	Biolegend	Cat#102102
anti-CD40 clone 1C10	Biolegend	Cat#102810
F(ab')2 goat anti-mouse IgG	ebioscience	Cat#16-1598-85

Bacterial and Virus Strains		
LCMV-WE	Obtained from F. Lehmann-Grube, Heinrich Pette Institut, University of Hamburg, Germany. Propagated in fibroblast cell line L929	Battegay 1991 (5)
Biological Samples		
Human peripheral blood	Healthy voluntary donors	
Chemicals, Peptides, and Recombinant Proteins		
ONX 0914	Provided by C.J. Kirk, Kezar Life Sciences, South San Francisco, USA	Muchamuel 2009 (6)
MG-132	Sigma Aldrich	Cat#M7449
PR-825	Provided by C.J. Kirk, Kezar Life Sciences, South San Francisco, USA	Muchamuel 2009 (6)
DMSO Hybri-Max™, sterile-filtered, BioReagent, suitable for hybridoma, ≥99.7%,	Sigma Aldrich	Cat#D2650
Cycloheximide	Sigma Aldrich	Cat#C1988-5G
^{35}S-cystein/methionine	Hartmann Analytik	Cat#IS-103
Phorbol 12-myristate 13-acetate (PMA)	Sigma Aldrich	Cat#P8139
Ionomycin calcium salt	Sigma Aldrich	Cat#I0634
ViaStain™ AO/PI Staining Solultions	Nexcelom Bioscience	Cat#CS2-0106-5mL
DAPI Fluoromount-G	SouthernBiotech	Cat#0100-20
Bovine Serum Albumin (BSA)	Sigma Aldrich	Cat#9647
Critical Commercial Assays		
Mouse IL-2 ELISA Ready-Set Go!®	ebioscience	Cat#88-1754-88
MACS® CD19 MicroBeads, mouse	Miltenyi	Cat#130-052-201
MACS® CD4 (L3T4) MicroBeads, mouse	Miltenyi	Cat#130-117-043
MACS® CD4+ T cell isolation kit, mouse	Miltenyi	Cat#130-104-454
MACS® CD14 MicroBeads, human	Miltenyi	Cat#130-050-201
MACS® CD4 MicroBeads, human	Miltenyi	Cat#130-045-101
Vybrant® MTT Cell Proliferation Assay Kit	ThermoFisher Scientific	Cat#V13154
FastStart DNA Master SYBR green-I kit	Roche	Cat#12239264001
RNeasy Mini kit	QIAGEN	Cat#74104
Deposited Data		

Experimental Models: Cell Lines		
T1	Provided by W. Schamel, University of Freiburg, Freiburg, Germany	Anjuère 1997 (7)
x63-m-IL2	Long-term stored, in house	Bubeník 1992 (8)
L929 fibroblast	Provided by F. Lehmann-Grube, Heinrich Pette Institut, University of Hamburg, Germany	Battegay 1991 (5)
CTLL-2	Provided by H. Haase, TU Berlin, Germany	ATCC® TIB-214™
Experimental Models: Organisms/Strains		
C57BL/6J (H-2^b)	Charles River	
LMP7$^{-/-}$	Provided by J.J. Monaco, Cincinnati Medical Center, Cincinannti, USA	Fehling 1994 (9)
LMP2$^{-/-}$		Van Kaer 1994 (10)
SMARTA (SM-1 x Ly5.1)	Provided by Swiss Immunological Mutant Mouse Repository	Oxenius 1998 (11)
DUSP6$^{-/-}$	Charles River	Maillet 2008 (12)
Oligonucleotides (5'-3' seqeuence)		
CCT TGG GCT GTG TTA ATA GTG G	MicroSynth	*cd69* fwd
GCT TCA GAA ACG TCA TGT CCT	MicroSynth	*cd69* rev
ACA TTG ACA CTT GTG CTC CT	MicroSynth	*il-2* fwd
TTC CTG TAA TTC TCC ATC CTG C	MicroSynth	*il-2* rev
CCC AAT AGT GCA ACG GAC TC	MicroSynth	*dusp6* fwd
GGG CTT CAT CTA TGA AAG AAA TGG	MicroSynth	*dusp6* rev
TGA AGG CAT CAA CAT TTC TGG	MicroSynth	*rpl13a* fwd
GGT AAG CAA ACT TTC TGG TAG G	MicroSynth	*rpl13a* rev
GTA CTT TAC AGA CAT GAT GCC TC	MicroSynth	*ipo8*: fwd
AAT GAA TAG CGG GAT ACA CTG	MicroSynth	*ipo8*: rev
Recombinant DNA		
Software and Algorithms		

GraphPad Prism 6	GraphPad Software, Inc.	
FACSDIVA Software	BD Biosciences	
Accuri C6 Software	BD Biosciences	
FlowJo V10	FLOWJO, LLC	
ImageJ Fiji	https://fiji.sc/	Schindelin 2012 (13)
Intensity Ratio Nuclei Cytoplasm Tool (modified!)		http://dev.mri.cnrs.fr/projects/imagej-macros/wiki/Intensity_Ratio_Nuclei_Cytoplasm_Tool and this paper (modified version)
Zeiss Zen 2011	Carl Zeiss	

Supplementary Materials

Buffer Name	Recipe
FACS buffer	PBS, 2% FBS, 2 mM EDTA, 2 mM NaN_3
PERM buffer	FACS-buffer + 0.1% Saponin
SDS sample buffer	In 26 ml for 3x: 8 ml glycerol, 0.4 ml Tris-pH 7, 6 ml 20% SDS
Whole cell lysis buffer (also used for lysis of nuclear extracts)	1% NP-40, 137 mM NaCl, 1 mM EDTA, 20 mM Tris-HCl, pH 7.2 at 25°C, 2 mM Na_3VO_4, 0.15% SDS, 1x PhosStop (Roche), 1x complete protease inhibitors (Roche), 0.1% sodium deoxycholate
Cytosolic extraction buffer	5 mM HEPES, 75 mM NaCl, 2.5 mM KCl, 0.5 mM $MgCl_2$, pH 7.4, 0.1% NP-40, 1x PhosSTOP Roche)
TBS-T	Tris-buffered saline (per L for 10x: 24.8 g Trizma base, 80 g NaCl pH 7.6), 0.15% Tween-20
NET-TON	50 mM TrisHCl, pH 8.0, 150 mM NaCl, 5 mM EDTA, 0.5% Triton X-100
NET-T	NET-TON with 650 mM NaCl

Cell Culture Media (Supplier)	Supplements
RPMI 1640 (Gibco)	+10% FBS, 1% pen-strep, 50 µM 2-mercaptoethanol
AIM-V (Gibco)	2% human serum, 1% pen-strep, 50 µM 2-mercaptoethanol

Disposables	Supplier
Protran 0.45 NC Premium nitrocellulose membrane	GE Healthcare
Whatman 3MM-CHR	GE Healthcare
Cellstar Cell Culture Plates, sterile with lid	Greiner bio one

Choice of sample size and statistical tests:

Figure 1 A and B) The first result showing reduced CD69 up-regulation was found on a different flow cytometer than the later experiments. Analysis of LMP2-deficient mice was conducted either together with WT control alone or with WT and LMP7-deficient mice. Therefore sample size n differs, but has a minimum of n = 3. As different flow cytometers were used non-normalized data has technical data variation violating the assumption of random sampling for analysis. In order to report all relevant results and fully subject them into analysis, the ratio between ONX 0914 treated and DMSO treated was used as pairs from each experiment thereby accounting for technical inter-experiment variation. Thus, all data could be subjected to analysis together via one-sample t-test.
Figure 1 C) In four independent experiments the fold up-regulation of IL-2 mRNA and CD69 mRNA showed high variation, but this could not be unambiguously accounted to technical variation. Therefore, the original untransformed data (normalized to unstimulated 0 h DMSO controls) was subjected to analysis as paired data from each experiment. Based on a 50% reduction at surface marker level in Figure 1A, a putative 50% reduction at mRNA level was *a priori* calculated using G-Power with input values: mean group 1: 10; mean group 2: 5, SD of both groups: 2, reaching effect size 2.5. Thus, to reach a minimum power of 0.8 at a confidence level of 95% a sample size of n = 4 was calculated for a matched-pairs two-tailed t-test.

Figure 2: We expected that the effect could be milder *in vivo* and calculated sample sizes *a priori* for the animal research proposal G-16/154 for a putative difference of 30% between ONX 0914 and vehicle. The approval accounted 6 mice per group. Preliminary tests with 2 mice only (as approved by the proposal) indicated that smaller variation could be expected than assumed before. We reduced the number of mice to 5 per group and the minimum of 3 per group in uninfected mice to reduce the amount of mice needed for animal experiments as demanded by ethical considerations. One mouse was not successfully infected with LCMV and was therefore not subjected to analysis (Vehicle, LCMV, median CD69 FI: 3511). Residual mice were subjected to analysis by two-way ANOVA, Sidak's post test.
Figure 3 D) Quantitative Immunoblot analysis from near-IR detections is internally normalized to tubulin loading control in our experiments. Thus, normalization was obtained with signals from the same detection instead of detecting total ERK after stripping. Total ERK was analyzed independently in blots or in flow cytometry. Re-use of antibody-solutions and blotting buffers as commonly performed in SDS-PAGE and western blotting creates technical data variation. The individual data points generated in reproductions of the experiment therefore cannot be assumed to be random samples all representing the population with equal chance. To account for this, the ratio of ONX 0914- or MG-132-treated over DMSO-treated was used from each individual experiment thus accounting for technical inter-experiment variation. Analysis was thus performed as one sample t-test with $\mu_0=1$. Sample size was calculated after observation of the first experiment yielding 15% difference. To assure a minimum power of 0.8 we calculated n = 5 using G-Power and then used the same expectations as *a priori* parameters for p-MEK and flow cytometric p-ERK analysis in Figure 3E.
Figure 3 E) Analysis and sample size as outlined for Figure 1A and Figure 3D.
Figure 3 F and G) Primary human CD4+ T cells were analyzed in three experiments using identical experimental conditions and flow cytometry settings as well as devices. Therefore, plain original data was subjected to analysis using two-way repeated measures ANOVA, Sidak's post test. Sample size n = 3 was calculated for a 50% difference expected based on Figure 1A) and a putative 15% SD using G-Power at a power level 0.8 and alpha error 0.05. For immunoblot analysis the same cells

were used and statistical evaluation was performed as in Figure 3D.
Figure 5B: Three independent experiments were performed and evaluated *post hoc*. Matched-pairs from simultaneous detections were subjected to paired t-tests to account for technical experiment-to-experiment variation.
Figure 5F: *Dusp6* mRNA analysis was performed in three independent experiments and analyzed as fold-change over DMSO unstimulated control. Two-way repeated measures ANOVA, Sidak's post test.

Supplementary Method Details:

Ex vivo expansion of CD4+ T cells

For *ex vivo* expansion 1x10^6 cells/ml were stimulated with 50 ng/ml phorbol-12-myristate-13-acetate (PMA) and 500 ng/ml ionomycin for 25 h, followed by cultivation in the presence of 40 U/ml recombinant m-IL-2 (produced with x63-m-IL2 cells (8), activity determined by MTT-assay with CTLL-2 cells and IL-2 ELISA; CTLL-2 were kindly provided by Hajo Haase, TU Berlin, Germany). IL-2 medium was renewed on day 4. Cells were either kryo-conserved on day 6 (RPMI 1640, 10% DMSO, 20% FBS) or used directly on day 7 for experiments. Kryo-conserved cells were thawed 16-20 h before use and kept overnight in medium containing 30 U/ml IL-2. Cell viability was checked by trypan blue or AO/PI staining before experiments (Nexcelom Bioscience).

Confocal Microscopy

Microscopic images were obtained with a Zeiss LSM 880 Axio Observer confocal microscope (Plan-Apochromat 63x/1.40 Oil DIC M27) at 1AU. Microscope settings for the channels of laser line 405 and 633 are shown in the table below:

Microscopy settings for each experiment	Total ERK1/2	p-ERK1/2
Laser Intensity	405: 0.2 %<br>633: 12 %	405: 0.2 %<br>633: 24 %
Master Gain	405: 620<br>633: 750	405: 620<br>633: 750
Digital Offset	405: 311<br>633: 820	405: 311<br>633: 810
Emission	405: 459 nm<br>633: 679 nm	405: 459 nm<br>633: 679 nm
Detection	405: 410-585 nm<br>633: 638-755 nm	405: 410-585 nm<br>633: 638-755 nm

In each experiment, 4-5 images per condition were obtained containing 30 – 50 cells per image. Nuclear intensities of p-ERK and total ERK-signals in microscopic images were quantified using Fiji. The Intensity Ratio Nuclei Cytoplasm Tool (http://dev.mri.cnrs.fr/projects/imagej-macros/wiki/Intensity_Ratio_Nuclei_Cytoplasm_Tool) was modified to obtain average nuclear intensity values using area overlay with the DAPI signal. The modified macro is available online as supplementary material. The median of image average intensities from the 4-5 images within one experiment was used to test statistical significance of the effect over three independent experiments and is represented as data points in bar graphs.

Radioactive labeling and immunoprecipitation

T1 cells (0.5–1x10^5/ml) were cultured with 200 U/ml recombinant m-IFN-y (Peprotech) for 3 days to induce higher IP expression. Consequently, 4-5x10^6 cells per sample were pre-treated for 2 h with ONX 0914, DMSO or MG-132, followed by activation with plate-bound antibodies against CD3 and CD28 for 2-3 h in the presence of MG-132 or DMSO. Medium was exchanged to cysteine/methionine-free medium containing dialyzed FBS for 1h starvation followed by a 15 min radioactive pulse (250 µCi/ml, 500 µl/sample) of ^{35}S-labeled cysteine/methionine (Hartmann Analytic, IS-103) added to starvation medium. Cells were washed with RPMI 1640 and chased 0 min, 20 min and 40 min after pulse. Medium was discarded and cells lysed in ice-cold whole cell lysis buffer. Total ^{35}S-incorporation was assessed via β-counts and used for loading normalization for DUSP6-immunoprecipitation performed with rabbit anti-DUSP6 (Abcam, ab220811, 30 µl Protein A EZview Affinity Gel, Sigma) for 5-6 h at 4°C. After washing (2x NET-TON, 1 x NET-T) the precipitate was boiled in SDS sample buffer for 5 min at 95°C. After SDS-PAGE autoradiography was performed in a phosphoimager (BioRad).

Supplementary References

1. Khan S, van den Broek M, Schwarz K, de Giuli R, Diener P a, Groettrup M. Immunoproteasomes largely replace constitutive proteasomes during an antiviral and antibacterial immune response in the liver. *J Immunol* (2001) **167**:6859–68. Available at: http://www.ncbi.nlm.nih.gov/pubmed/11739503

2. Guillaume B, Chapiro J, Stroobant V, Colau D, Van Holle B, Parvizi G, Bousquet-Dubouch M-P, Théate I, Parmentier N, Van den Eynde BJ. Two abundant proteasome subtypes that uniquely process some antigens presented by HLA class I molecules. *Proc Natl Acad Sci U S A* (2010) **107**:18599–604. doi:10.1073/pnas.1009778107

3. Kremer M, Henn A, Kolb C, Basler M, Moebius J, Guillaume B, Leist M, Van den Eynde BJ, Groettrup M. Reduced immunoproteasome formation and accumulation of immunoproteasomal precursors in the brains of lymphocytic choriomeningitis virus-infected mice. *J Immunol* (2010) **185**:5549–60. doi:10.4049/jimmunol.1001517

4. Macagno A, Gilliet M, Sallusto F, Lanzavecchia A, Nestle FO, Groettrup M. Dendritic cells up-regulate immunoproteasomes and the proteasome regulator PA28 during maturation. *Eur J Immunol* (1999) **29**:4037–4042. doi:10.1002/(SICI)1521-4141(199912)29:12<4037::AID-IMMU4037>3.0.CO;2-T

5. Battegay M, Cooper S, Althage A, Bänziger J, Hengartner H, Zinkernagel RM. Quantification of lymphocytic choriomeningitis virus with an immunological focus assay in 24- or 96-well plates. *J Virol Methods* (1991) **33**:191–198. doi:10.1016/0166-0934(91)90018-U

6. Muchamuel T, Basler M, Aujay M a, Suzuki E, Kalim KW, Lauer C, Sylvain C, Ring ER, Shields J, Jiang J, et al. A selective inhibitor of the immunoproteasome subunit LMP7 blocks cytokine production and attenuates progression of experimental arthritis. *Nat Med* (2009) **15**:781–7. doi:10.1038/nm.1978

7. Anjuère F, Kuznetsov D, Romero P, Cerottini JC, Jongeneel C V, Luescher IF. Differential

roles of T cell receptor alpha and beta chains in ligand binding among H-2Kd-restricted cytolytic T lymphocyte clones specific for a photoreactive Plasmodium berghei circumsporozoite peptide derivative. *J Biol Chem* (1997) **272**:8505–14. doi:10.1074/jbc.272.13.8505

8. Bubeník J, Lotzová E, Indrová M, Šímová J, Jandlová T, Bubeníková D. Use of IL-2 gene transfer in local immunotherapy of cancer. *Cancer Lett* (1992) **62**:257–262. doi:10.1016/0304-3835(92)90104-4

9. Fehling HJ, Swat W, Laplace C, Kühn R, Rajewsky K, Müller U, von Boehmer H. MHC class I expression in mice lacking the proteasome subunit LMP-7. *Science* (1994) **265**:1234–7. Available at: http://www.ncbi.nlm.nih.gov/pubmed/8066463

10. Van Kaer L, Ashton-Rickardt PG, Eichelberger M, Gaczynska M, Nagashima K, Rock KL, Goldberg a L, Doherty PC, Tonegawa S. Altered peptidase and viral-specific T cell response in LMP2 mutant mice. *Immunity* (1994) **1**:533–41. Available at: http://www.ncbi.nlm.nih.gov/pubmed/7600282

11. Oxenius a, Bachmann MF, Zinkernagel RM, Hengartner H. Virus-specific MHC-class II-restricted TCR-transgenic mice: effects on humoral and cellular immune responses after viral infection. *Eur J Immunol* (1998) **28**:390–400. doi:10.1002/(SICI)1521-4141(199801)28:01<390::AID-IMMU390>3.0.CO;2-O [pii]

12. Maillet M, Purcell NH, Sargent MA, York AJ, Bueno OF, Molkentin JD. DUSP6 (MKP3) null mice show enhanced ERK1/2 phosphorylation at baseline and increased myocyte proliferation in the heart affecting disease susceptibility. *J Biol Chem* (2008) **283**:31246–31255. doi:10.1074/jbc.M806085200

13. Schindelin J, Arganda-Carreras I, Frise E, Kaynig V, Longair M, Pietzsch T, Preibisch S, Rueden C, Saalfeld S, Schmid B, et al. Fiji: an open-source platform for biological-image analysis. *Nat Methods* (2012) **9**:676–82. doi:10.1038/nmeth.2019

Supplementary Figure Legends

Figure S1 (Related to Figure 1): IL-2 supplementation does not rescue proliferation / Expanded CD4+ T cells are affected by ONX 0914 treatment.

A CFSE-dilution profile of primary murine MACS-enriched CD4+ T cells that were activated with plate-bound anti-CD3/anti-CD28 antibodies for 72 h in the presence or absence of different ONX 0914 concentrations and either with (right) or without (left) supplementation of recombinant murine IL-2.

B Immunoblot analysis of proteasome subunits β5c, LMP7 and β1c in primary naive CD4+ T cells isolated from WT or LMP7-deficient mice compared to *ex vivo* expanded CD4+ T cells. Tubulin was used as a loading control.

C Representative CD69 expression profiles and MFI quantification (triplicate measurement) of anti-CD3/anti-CD28 re-stimulated (5 h), expanded CD4+ T cells after 2 h pulse-treatment with DMSO or

ONX 0914 before activation. Scatter-plot shows the ratio of ONX 0914-treated over DMSO treated cells from four independent experiments. Mean+95% CI, one-sample t-test, horizontal dashed line indicates null hypothesis value $\mu_0 = 1$.

Figure S2 (Related to Figure 3): ERK-phosphorylation, but not total ERK and not IκBα are affected by ONX 0914

A Expanded CD4+ T cells from WT or LMP7$^{-/-}$ mice were pulse-treated with DMSO or ONX 0914 for 2 h, followed by activation with plate bound anti-CD3/anti-CD28 antibodies for indicated time periods. Lysates were analyzed by ECL-based immunoblots and quantified with densitometry normalized to tubulin loading control. Vertical grey line indicates signals were from the same membrane and detection, but not originally juxtaposed. Bar graphs show quantification of normalized signals (to highest value) and analysis with two-way ANOVA, Sidak's post test. Three independent reproductions for WT and two reproductions for LMP7$^{-/-}$ samples are shown (mean ± SD)

B MACS enriched naive CD4+ T cells from WT or LMP7$^{-/-}$ mice were pulse-treated for 2 h with DMSO or ONX 0914 followed by activation with anti-CD3/anti-CD28 antibodies and flow cytometric analysis of intracellular ERK protein. A representative example of two independent experiments is shown.

C Naive T cells isolated from WT spleen were pulse-treated for 2 h with 300 nM ONX 0914 or 0.3% DMSO followed by activation with anti-CD3/anti-CD28 antibodies for indicated time periods. IκBα content was measured by intracellular flow cytometry and median fluorescence intensity with SD from triplicates is shown.

D Experiment as in **C** but with PMA/ionomycin stimulation and continuous MG-132 treatment as control.

E Expanded murine CD4+ T cells were pulse-treated with DMSO or ONX 0914 for 2 h before activation with PMA and ionomycin for indicated time periods. IκBα degradation was detected using immunoblotting with α-tubulin as a loading control

Figure S3 (Related to Figure 4): Ubiquitin-conjugates are not detected in Molt4 cells / LMP7-deficient cells do not show ubiquitin-conjugates or DUSP dysregulation

A Effect of ONX 0914 treatment on ubiquitin conjugate accumulation in Molt 4 cells. Experiment performed as detailed in Muchamuel *et al.* 2009, Nature Medicine, 15;7, 781-7, Supplementary Figure S1A. One example of two experiments.

B Expanded CD4+ T cells from WT or LMP7-deficient mice were pulse-treated for 2 h with 0.3% DMSO (D) or 300 nM ONX 0914 (X) and consequently activated with plate bound anti-CD3/anti-CD28 antibodies for indicated time periods. Immunoblot analysis as indicated. One example of two experiments.

C Naive MACS-enriched CD4+ T cells from WT or LMP7-deficient mice were treated as in B, with 10 µM MG-132 as additional control in the 5 h stimulated time point. One example of three independent experiments.

Figure S4 (Related to Figure 4 and 5): T1 cells serve as a model cell line similarly affected by ONX 0914 as primary T cells

A T1 cells pre-treated with 200 U/ml IFN-γ for 3 days were then pre-treated with DMSO or ONX 0914 for 2 h before activation with plate-bound anti-CD3/CD28 antibodies for 6 h. IL-2 in the supernatants was measured by ELISA. One representative example of three independent experiments

is shown as mean ±SD from triplicates.
B T1 cells as in **A** and additional continuous MG-132-treated group were activated and immunoblots performed as indicated. One example of three independent experiments is displayed.
C LMP7 and β5c content of T1 cells after three days of IFN-γ treatment. IOTA and γ-tubulin were used as loading controls. Shown is one example of three independent experiments with similar outcome.
D T1 cells treated as in **B** were used for RNA extraction and q-RT-PCR after 3 h of activation. Fold up-regulation of *Dusp6* transcripts over DMSO unstim control is shown. Data were pooled from three independent experiments (mean ± SD). Repeated-measures ANOVA, Sidak's post test, p-values as indicated.
E T1 cells as in **B** were activated for indicated time periods and immunoblots were performed for indicated proteins. γ-tubulin was used as loading control. One example out of two independent experiments is shown.

Figure S5: ERK-phosphorylation, but not nuclear translocation of ERK is affected by ONX 0914
A Expanded CD4+ T cells were pre-treated with 0.3% DMSO, 300 nM ONX 0914 or 10 μM MG-132 for 2 h before activation with plate-bound anti-CD3/anti-CD28 antibodies for 3 h (MG-132 was given continuously). Cells were stained against p-ERK1/2 and DAPI and confocal images were obtained with a Zeiss LSM 880 microscope. Average nuclear intensities were quantified in 4-5 images per experiment in Fuji with a macro. Median values of averages from three independent experiments were pooled for analysis (bar graphs, mean ± SD of n = 3). Repeated-measures ANOVA, Sidak's post test, p-values as indicated.
B Experiment as in **A**, but with staining against total ERK1/2.

Figure S6: Effects on T cell activation by ONX 0914 are not attributed to DUSP6 alone
A, B MACS enriched CD4+ T cells from WT ($DUSP6^{+/+}$) and $DUSP6^{-/-}$ mice were pulse-treated for 2 h with 300 nM ONX 0914 or 0.3% DMSO before activation with plate-bound anti-CD3/anti-CD28 antibodies for 5 h. Cells were harvested and analyzed by flow cytometry. Representative blots for gating (upper panel) and histograms (lower panel) from one experiment are shown in **A**. Quantification of median fluorescence intensities as pooled data from three independent experiments is shown in **B** (mean ± SD). Two-way ANOVA, Sidak's post test, p-values as indicated.
C, D Supernatants from cells treated as in **A** were used to quantify IL-2 secretion by ELISA. One representative example (mean ± SD from triplicates) of three independent experiments is shown in **C**. Relative reduction of IL-2 secretion after ONX 0914 treatment is shown as ratio of ONX 0914-treated over DMSO-treated cells from three independent experiments in **D**. One-sample t test with null hypothesis mean value $\mu_0 = 1$ (dashed line), p-values as indicated, mean with 95% CI. Comparison between $DUSP6^{+/+}$ and $DUSP6^{-/-}$ was performed as paired t test, p-values are indicated in the figure.
E, F MACS enriched CD4+ T cells from WT ($DUSP6^{+/+}$), heterozygous ($DUSP6^{+/-}$) and $DUSP6^{-/-}$ mice were pulse-treated for 2 h with 300 nM ONX 0914 or 0.3% DMSO before activation with plate-bound anti-CD3/anti-CD28 antibodies for 3 h. Cells were harvested and stained for intracellular p-ERK1/2 for analysis in flow cytometry. One representative example of histograms is shown in **E**. Ratios of p-ERK1/2 MFI of pERK+ CD4+ cells between ONX 0914-treated over DMSO-treated cells is shown in **F**. Graphs represent pooled data from three independent experiments (mean with

95% CI). One sample t tests with null hypothesis mean value $\mu_0 = 1$ (dashed line), p-values as indicated in the figure.
G Immunoblot analysis of indicated proteins in splenocytes after CD4+ sort, liver homogenate or purified CD4+ T cells from WT, DUSP6$^{+/-}$ or DUSP6$^{-/-}$ mice to confirm knockout or heterozygosity for DUSP6 at protein level. One example of two experiments.

Figure S7 (Related to Figure 6): Immunoproteasome inhibition impairs B cell activation
A Primary murine B cells were purified from spleen by MACS, pulse-treated with DMSO or ONX 0914 for 2 h or continuously treated with MG-132 before activation with F(ab')2 and anti-CD40 for 6 h. Up-regulation of CD69 on the surface of CD19+ cells was measured by flow cytometry (histogram, left) and quantified as shown in the bar graph on the right. Graph shows mean +SD from duplicate measurements. Colors and assignment in the histogram (left panel) correspond to colors and assignments in the bar graph (right panel). Shown is one example of three independent experiments with similar outcome.
B CD69 up-regulation was quantified in three independent experiments as shown in **A** after 5 - 6 h of activation with F(ab')2 and anti-CD40. Ratio of compound-treated over DMSO control is shown from three experiments as mean with 95% CI. One sample t-tests, p-values indicated in the figure.
C Murine CD19+ B cells were pre-treated with ONX 0914 or DMSO for 2 h before activation with PMA/ionomycin for 5 h. CD69 up-regulation was measured by flow cytometry. One example of two independent experiments with similar outcome is shown (mean + SD of duplicate measurements).
D Cells treated as in **A** and **B** were activated for indicated time periods and immunoblot analysis was performed for indicated proteins in whole cell lysates. One example of two independent experiments with similar outcome is shown.

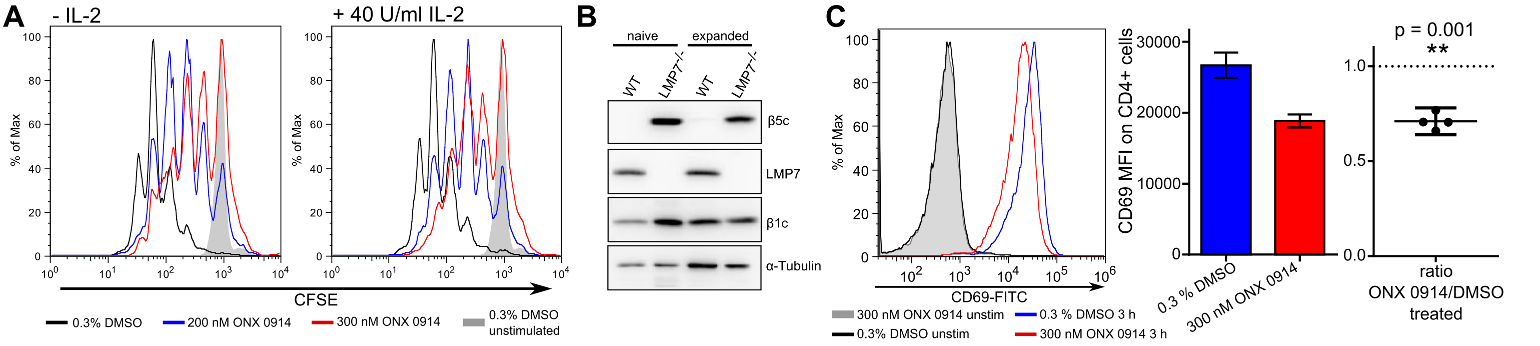

Schmidt *et al.*, 2018, Figure S1

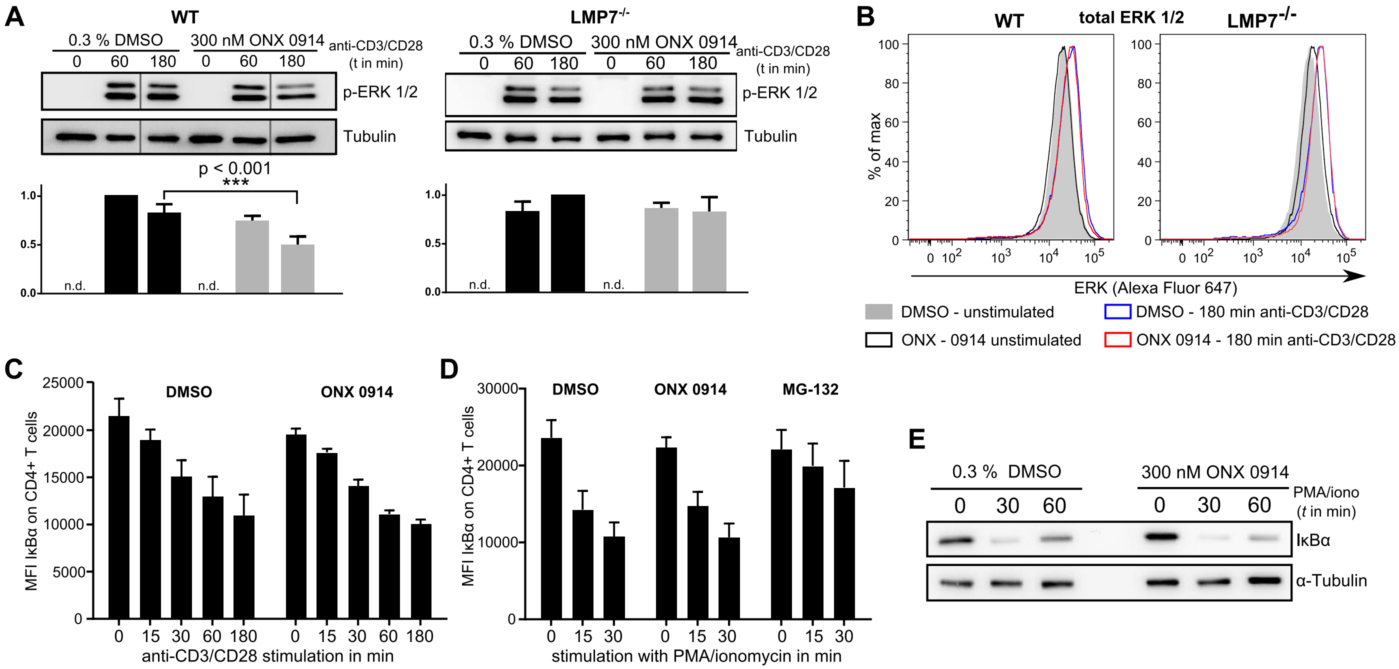

Schmidt *et al.*, 2018, Figure S2

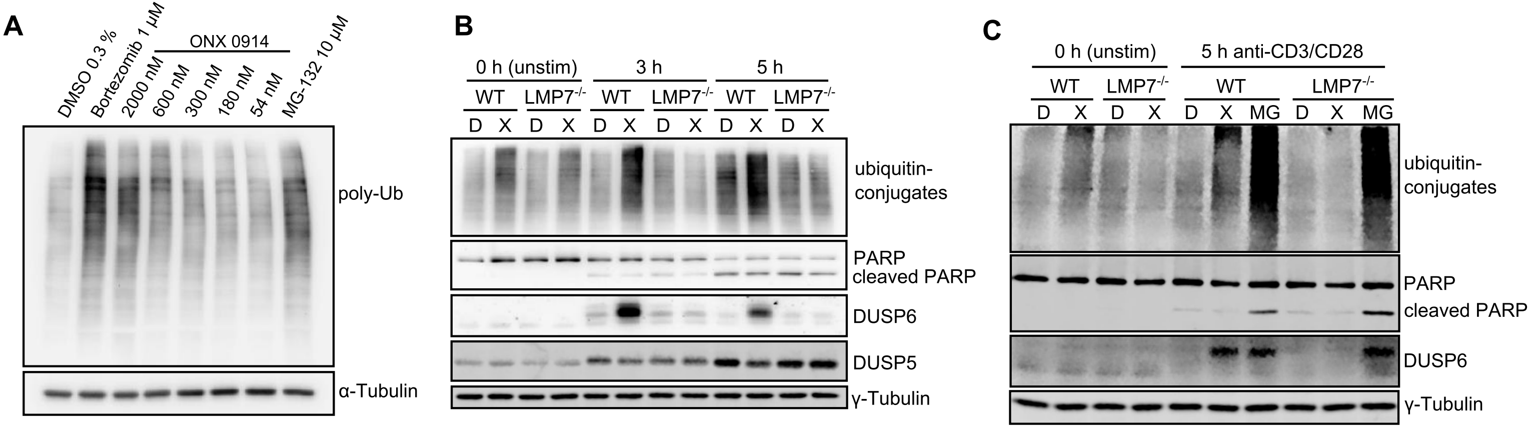

Schmidt *et al.*, 2018, Figure S3

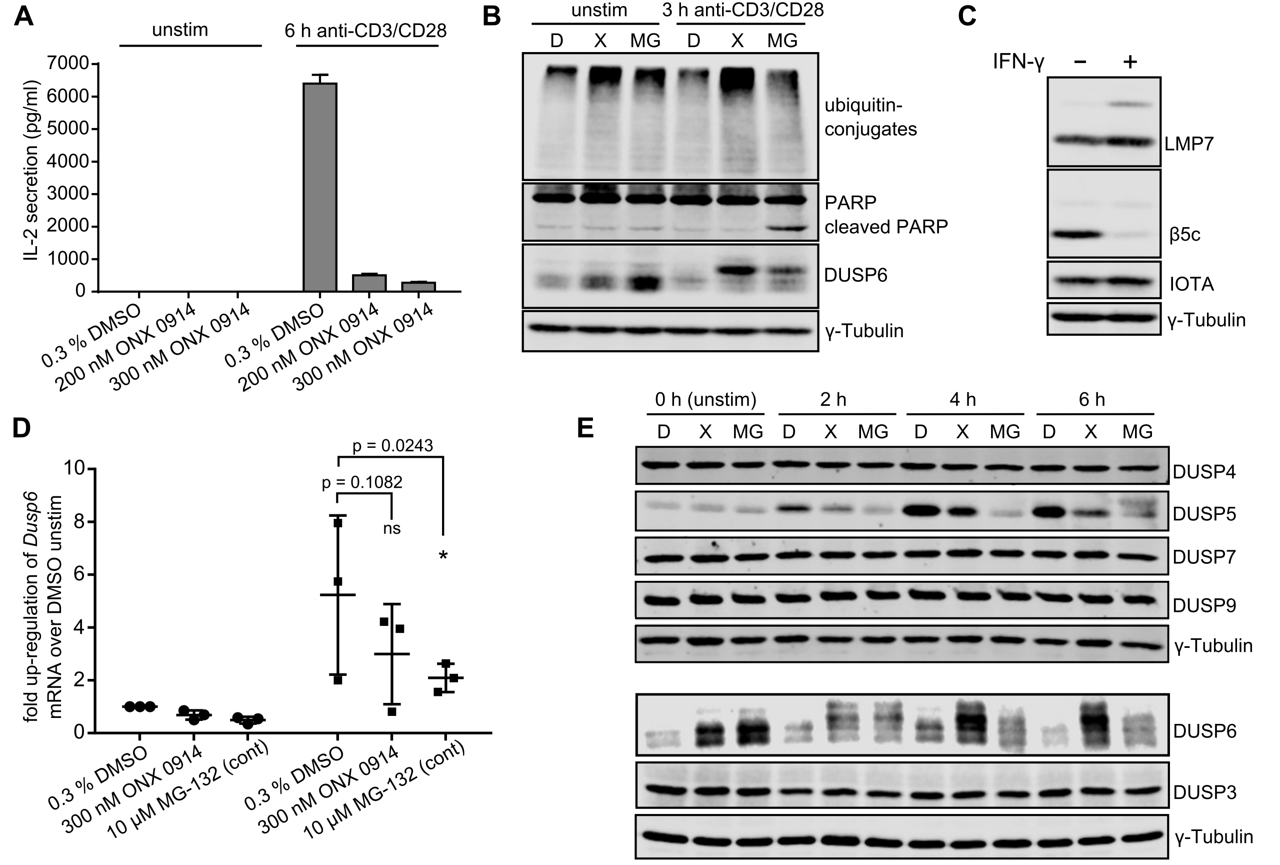

Schmidt *et al.*, 2018, Figure S4

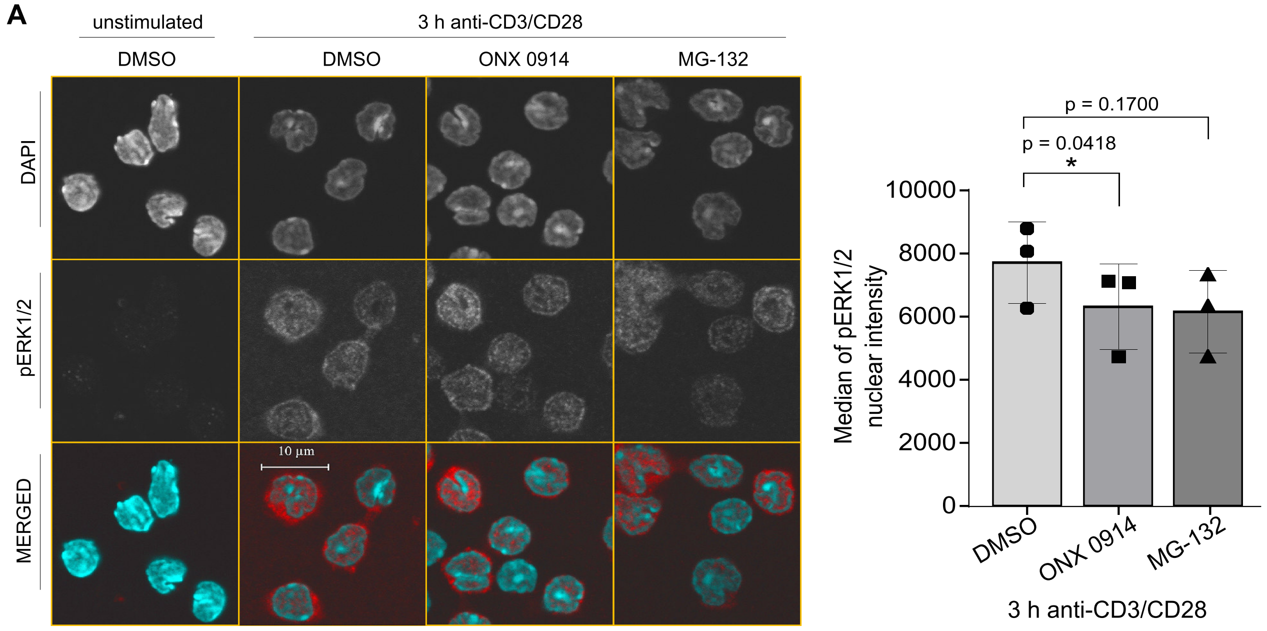

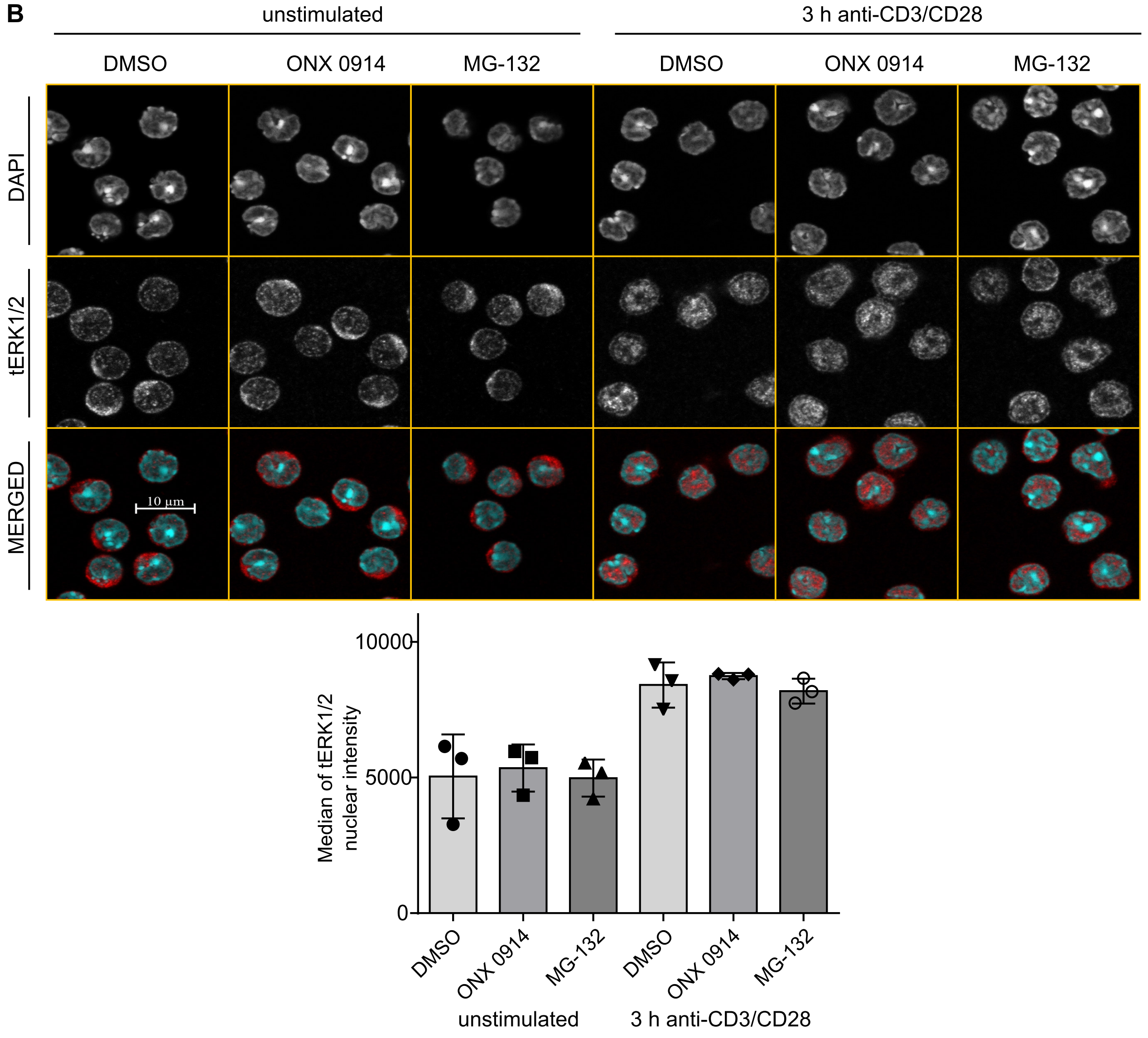

Schmidt *et al.*, 2018, Figure S5

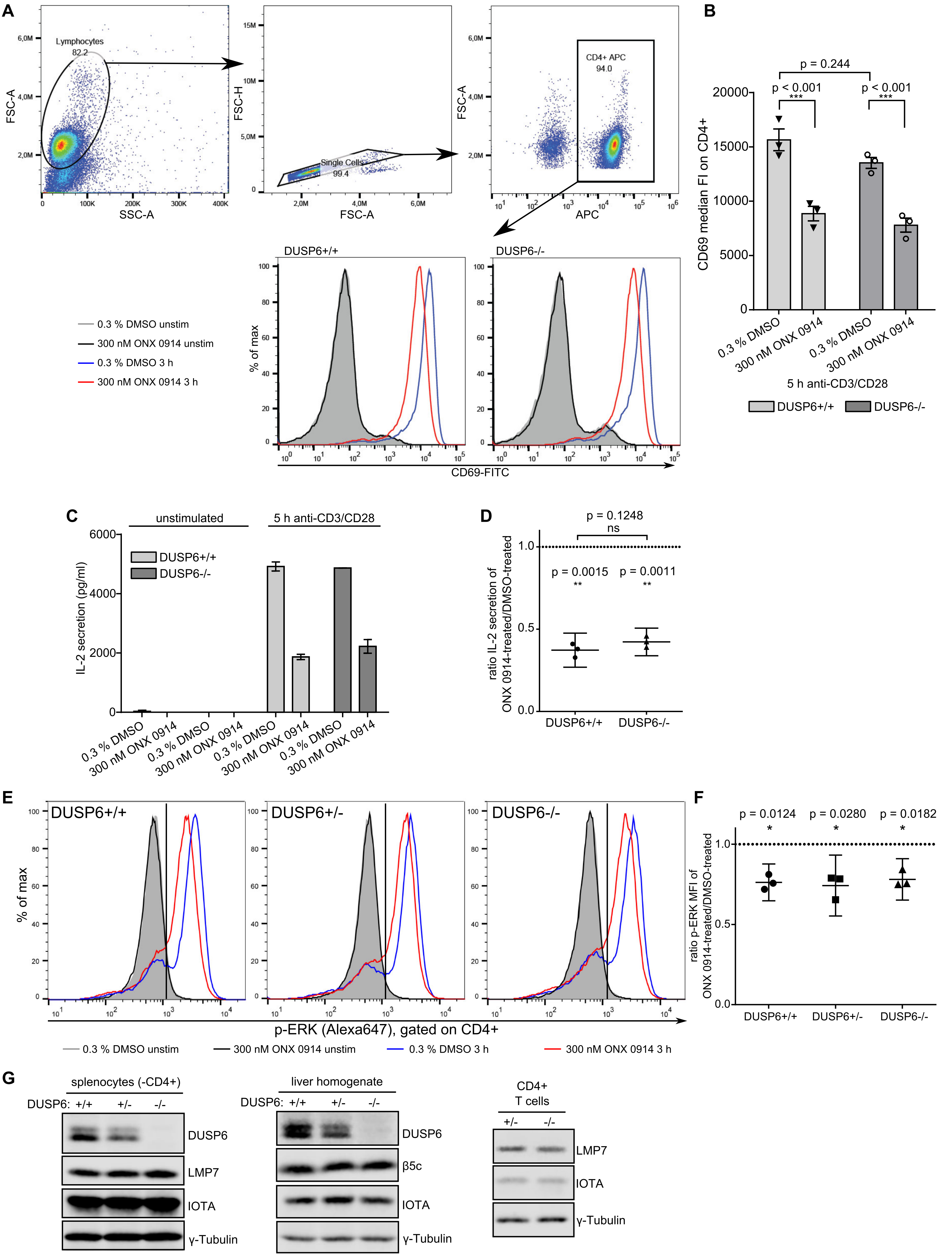

Schmidt *et al.*, 2018, Figure S6

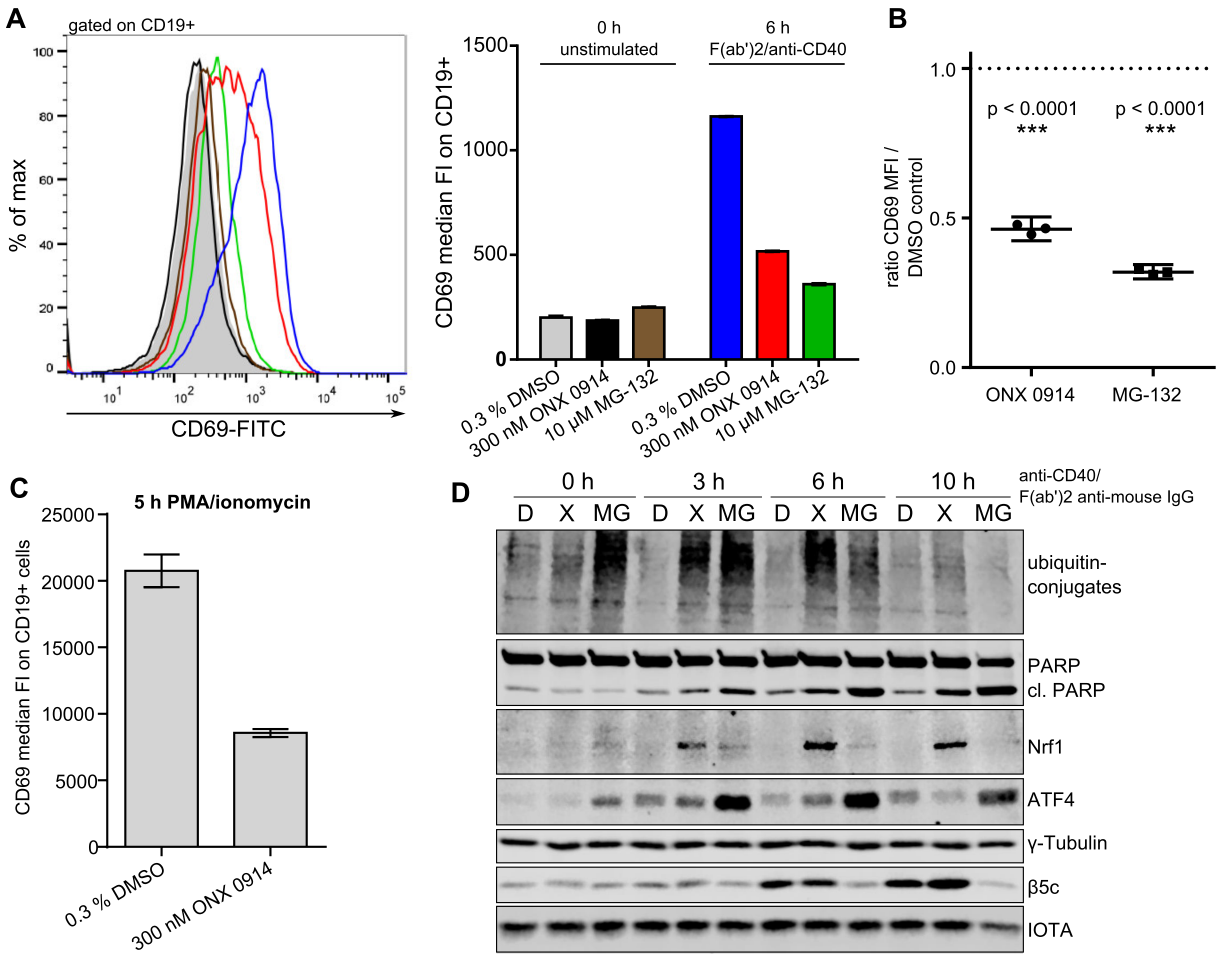

Schmidt *et al.*, 2018, Figure S7
